# DEEPOMICS FFPE, a deep neural network model, identifies DNA sequencing artifacts from formalin fixed paraffin embedded tissue with high accuracy

**DOI:** 10.1038/s41598-024-53167-0

**Published:** 2024-01-31

**Authors:** Dong-hyuk Heo, Inyoung Kim, Heejae Seo, Seong-Gwang Kim, Minji Kim, Jiin Park, Hongsil Park, Seungmo Kang, Juhee Kim, Soonmyung Paik, Seong-Eui Hong

**Affiliations:** Theragen Bio Co., Ltd., Seongnam, Gyeonggi-do 13488 Republic of Korea

**Keywords:** Cancer genomics, Genetic markers, Bioinformatics

## Abstract

Formalin-fixed, paraffin-embedded (FFPE) tissue specimens are routinely used in pathological diagnosis, but their large number of artifactual mutations complicate the evaluation of companion diagnostics and analysis of next-generation sequencing data. Identification of variants with low allele frequencies is challenging because existing FFPE filtering tools label all low-frequency variants as artifacts. To address this problem, we aimed to develop DEEPOMICS FFPE, an AI model that can classify a true variant from an artifact. Paired whole exome sequencing data from fresh frozen and FFPE samples from 24 tumors were obtained from public sources and used as training and validation sets at a ratio of 7:3. A deep neural network model with three hidden layers was trained with input features using outputs of the MuTect2 caller. Contributing features were identified using the SHapley Additive exPlanations algorithm and optimized based on training results. The performance of the final model (DEEPOMICS FFPE) was compared with those of existing models (MuTect filter, FFPolish, and SOBDetector) by using well-defined test datasets. We found 41 discriminating properties for FFPE artifacts. Optimization of property quantification improved the model performance. DEEPOMICS FFPE removed 99.6% of artifacts while maintaining 87.1% of true variants, with an F1-score of 88.3 in the entire dataset not used for training, which is significantly higher than those of existing tools. Its performance was maintained even for low-allele-fraction variants with a specificity of 0.995, suggesting that it can be used to identify subclonal variants. Different from existing methods, DEEPOMICS FFPE identified most of the sequencing artifacts in the FFPE samples while retaining more of true variants, including those of low allele frequencies. The newly developed tool DEEPOMICS FFPE may be useful in designing capture panels for personalized circulating tumor DNA assay and identifying candidate neoepitopes for personalized vaccine design. DEEPOMICS FFPE is freely available on the web (http://deepomics.co.kr/ffpe) for research.

## Introduction

Formalin fixation followed by paraffin embedding is universally practiced for routine clinical processing and storage of tissue samples because it allows thin sectioning for histopathology, immunohistochemistry, and in situ hybridization for companion diagnostics as well as long-term storage at room temperature. However, formalin-fixed, paraffin-embedded (FFPE) tissues are not ideal starting materials for molecular analyses, including next-generation sequencing (NGS). Formalin fixation leads to fragmentation of nucleic acids and hydrolytic deamination of cytosine^1–3^ The deamination of cytosine and 5-methylcytosine induces deoxyuridine(dU):G and T:G mismatches, respectively, eventually creating artificial C:G>T:A substitution^1–3^. The substitution could be also induced by heat treatment for the reversal of crosslinking when DNA is extracted from the blocks^1^. Suboptimal fixation and DNA/RNA extraction could affect the severity of the damage^4,5^. Despite these limitations, FFPE tissues are still the major starting materials for NGS performed as companion diagnostics to identify driver mutations. Currently used capture or amplicon-based targeted sequencing cancer panels are analytically validated for accurate variant calls for hotspot mutations with a limit of detection of 5% variant allele frequency. However, confident identification of subclonal driver mutations with low allele frequencies and accurate variant calling of whole-exome or whole-genome sequencing data remain huge challenges. In addition, emerging clinical applications of NGS, such as personalized circulating tumor DNA assay for minimal residual disease detection and personalized neoepitope targeted therapeutic vaccination, require accurate variant calls from non-hotspot mutations. Therefore, a robust method to filter FFPE-induced artificial variants from NGS data must be developed.

A simple strategy to reduce potential artifacts is ignoring all mutations with ≤ 5% allele frequencies (AFs) because FFPE-induced errors are randomly located across the genome and the AFs of artifacts might be lower than 5%^4^. However, this approach inevitably leads to the filtering of true subclonal mutations with clinical importance, such as the T790M mutation of the epidermal growth factor receptor (*EGFR*) gene^6^. Therefore, experimental procedures to improve the quality of nucleic acids from FFPE samples and minimize false positives must be optimized.

Experimental approaches have been suggested to minimize FFPE-induced artifacts. Uracil-DNA glycosylase (UDG) and thymine-DNA glycosylase remove deoxyuridine from dU:G mismatch and thymine from T:G mismatch, respectively, consequently generating abasic sites^7,8^. Template molecules harboring abasic sites are expected to be excluded from PCR amplification. Treatment with UDG can reduce C:G>T:A by 40–81%^8^. However, UDG preferentially cleaves a glycosidic bond in deoxyuridine in NdU[G/C] contexts compared with [A/T]dU[A/T] contexts (N represents any base nucleotide)^9^. FFPE-induced mutational signature is similar to SBS1 and SBS30, in which NC>TG and NC>TA contexts are predominant^4^. Thus, UDG treatment could ineffectively remove uracil in certain contexts. High-fidelity polymerase Pfu could be used to mitigate the risk of incorporating adenine base opposite to uracil^10^.

A bioinformatic approach can be applied for accurate variant calling. Genome Analysis ToolKit (GATK, https://gatk.broadinstitute.org/hc/en-us) offers tools for variant discovery, such as a somatic variant caller and a FFPE filter to remove false positives. The filter works based on the assumption that artifacts are generally strand biased. The existing tools described earlier have limited clinical application because they can remove either only a portion or most of the artifacts but at the cost of losing a significant portion of true variants.

We hypothesized that deep neural networks can be trained with paired FF-FFPE sequencing data to distinguish artificial FFPE-induced variants from true variants. In this study, we aimed to develop DEEPOMICS FFPE, a tool that can distinguish artifactual mutations in FFPE variant calls. This model is based on deep neural networks and has learned the characteristics of artifacts distinguishable from those of true variants. To evaluate the performance of the model comprehensively, we tested DEEPOMICS FFPE on FFPE exome sequencing data from various cancer types and variant calls with low mutation allele frequencies (1% < minor allele frequency, MAF < 5%). Our tool showed superior specificity, sensitivity, and F1-score over previously introduced tools. Specifically, DEEPOMICS FFPE identified more artifacts while preserving more true variants than other tools. We believe DEEPOMICS FFPE can provide a better variant pool from which clinicians can identify clinically important variants for accurately estimating tumor mutation burden (TMB), identifying neoepitopes, and characterizing tumor specific mutation signatures.

## Results

### DNAs from FFPE tissues are highly fragmented

To explore the characteristics of DNA extracted from FFPE tissues, we used publicly available whole-exome sequencing (WES) datasets^2,5,11^. These datasets are composed of WES data generated from FFPE tissues and matched fresh frozen (FF) tissues from five cancer types (2 lung cancers, 1 fibrosarcoma, 4 liver cancers, 4 colon cancers, and 13 breast cancers). Considering that these datasets were generated by three different groups, we believe they reflect various qualities of WES data resulting from variations in ischemia time before fixation, duration of formalin fixation, DNA extraction method, reverse-crosslinking method, and library preparation method.

**Figure 1:**
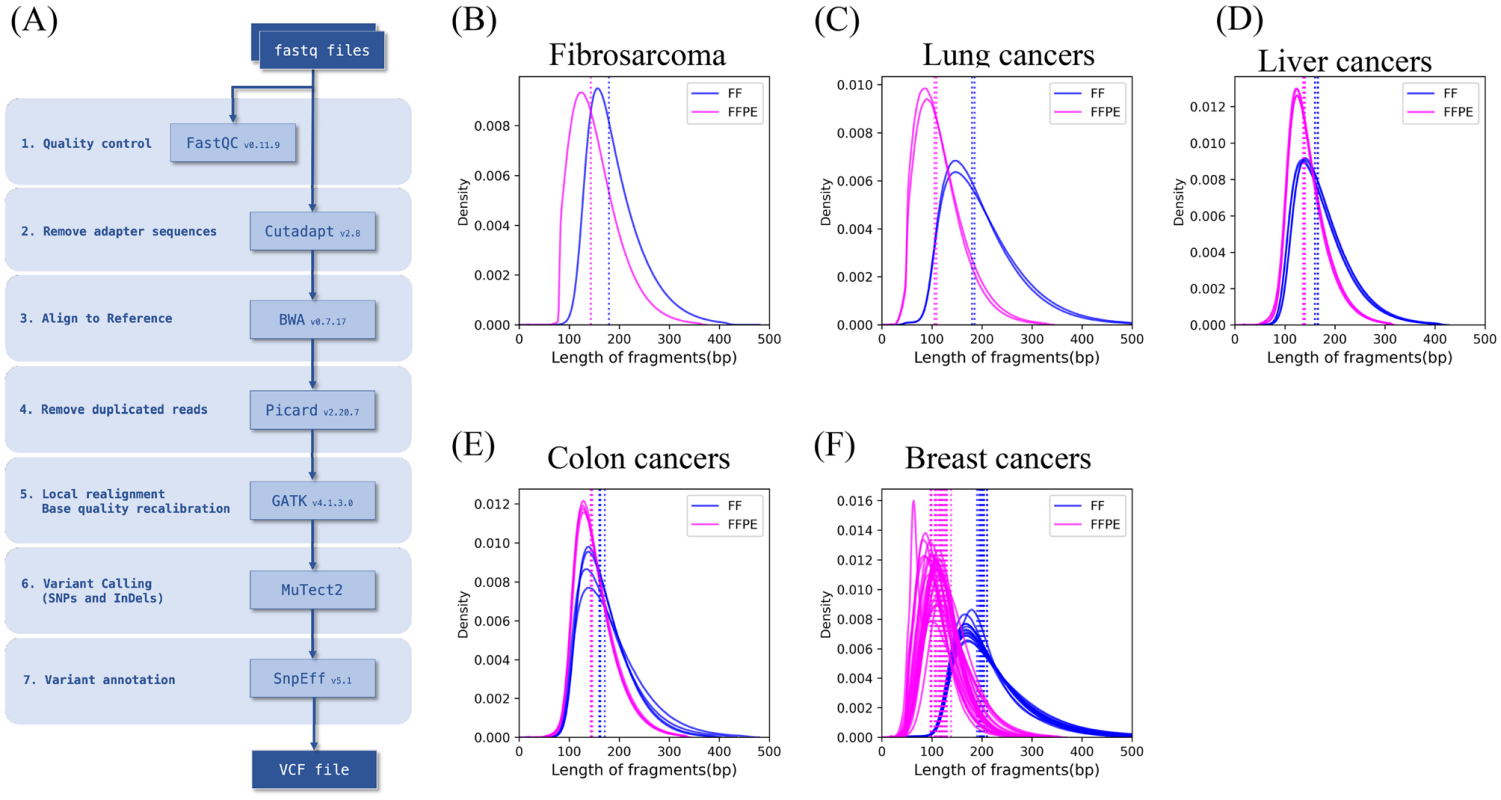
WES workflow and DNA qualities used in this study. Overview of WES workflow used in this study (**A**). The distribution of the length of insert fragment from FF (blue line) and FFPE (magenta line) were plotted for fibrosarcoma (**B**), lung cancer (**C**), liver cancer (**D**), colon cancer (**E**), and breast cancer (**F**). The vertical dotted lines indicate the median values for the length of insert from FF (blue line) and FFPE (magenta line).

In this study, we used a typical WES workflow (Fig. 1A, “Methods”). The insert fragments from the FFPE samples were shorter than the matched FF samples (Fig. 1B–F), suggesting that DNA from the FFPE samples was highly fragmented as previously reported^2,3^. The fragments from the breast and lung cancer FFPE samples were even shorter than those from the other cancer FFPE samples (Fig. 1C and F compared with B, D, E). This result indicated heterogeneity among the FFPE samples, supporting our assumption that these datasets reflect various DNA and WES data qualities.

### Characteristics of FFPE-induced artifacts

**Figure 2:**
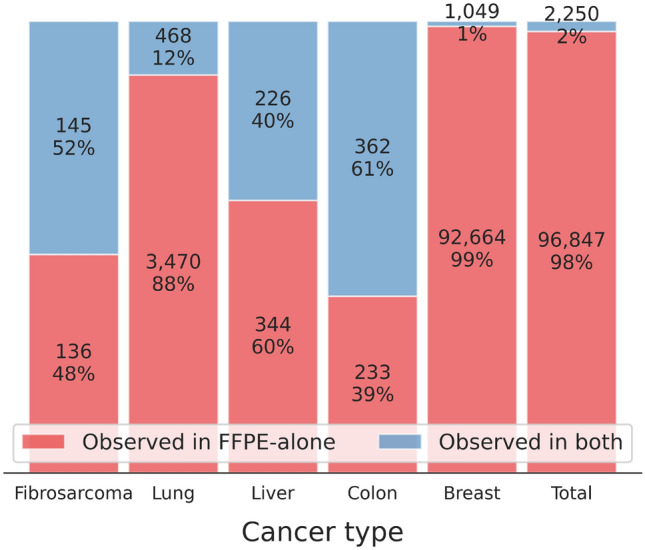
Proportion of artifactual mutations among variants observed in FFPE samples. Bar plot represents the percentage of the number of FFPE-artifacts in red and true variants in blue from the given cancer samples. The number of variant calls were indicated within the bars. X-axis indicates the cancer samples used in this study.

**Figure 3:**
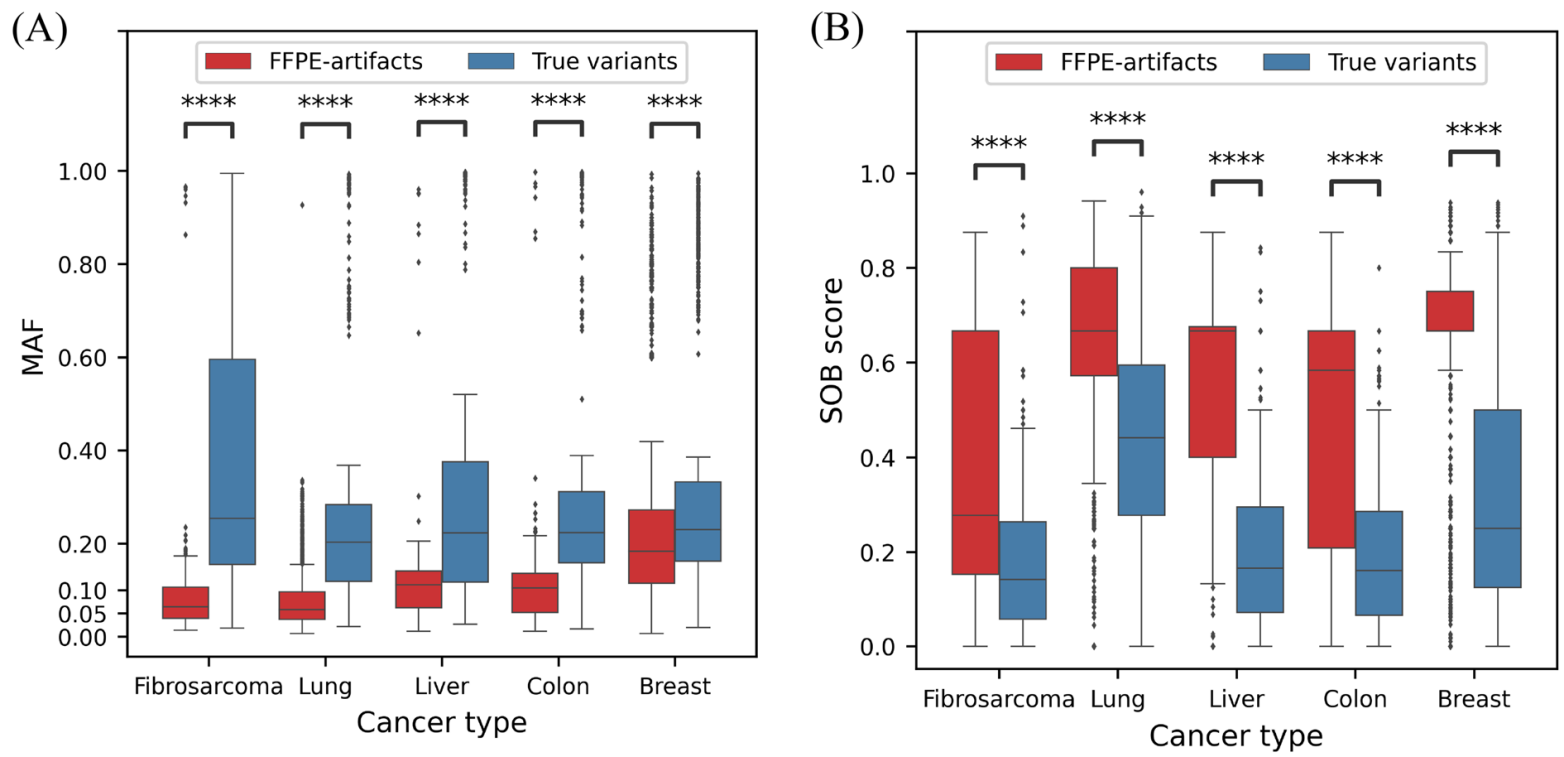
Characteristics of variants that were observed in FFPE samples. Mutation allele frequencies (MAF) (**A**) and SOB scores (**B**) were plotted for FFPE-artifacts in red and for true variants in blue. X-axis indicates the cancer samples used in this study. ****Denotes statistical significance (p-value < 0.0001, Mann–Whitney *U*-test).

**Figure 4:**
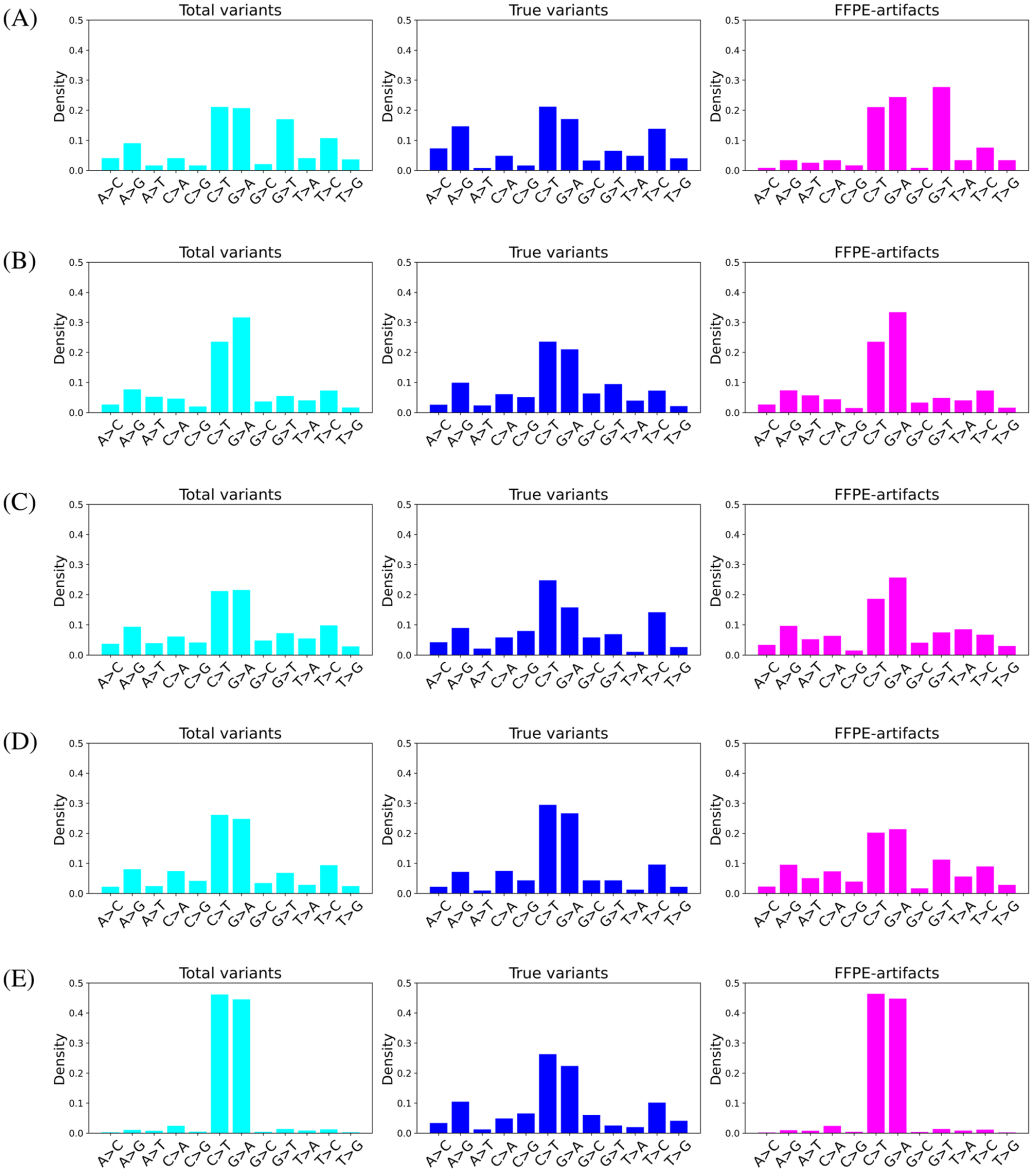
SNV types. The fractions of SNV type observed in FFPE samples were plotted for fibrosarcoma (**A**), lung cancer (**B**), liver cancer (**C**), colon cancer (**D**), and breast cancer (**E**). The SNV type of total variants (in the cyan plot, left) and true variants (in the blue plot, middle) and FFPE-artifacts (in the magenta plot, right) were shown.

GATK-MuTect2 is a reliable and widely used somatic variant calling toolkit^12–15^. After variant calling with GATK-MuTect2, we found that several variants in the FFPE samples were not present in the matched FF samples (Fig. 2). Specifically, approximately 80 times more variants were called in the FFPE samples than in the matched FF samples in breast cancers (Fig. 2), even though sequencing depth and coverage were compatible or even higher in the matched FF samples (Supplementary Table S1). This result implies that the FFPE-only variants could be FFPE-induced artifacts. Previous studies demonstrated that low allele frequency, strand bias, and predominant C:G>T:A substitutions are the main characteristics of FFPE-induced artifacts^2,4,6,16^. To assess the possibility, we stratified the variants called in FFPE into those called in FFPE-only and in both FFPE and FF, hereafter called “FFPE-artifacts” and “true variants”, respectively. We compared the characteristics between the FFPE-artifacts and true variants. The MAF of the FFPE-artifacts was lower than that of the true variants in all cancer types (Fig. 3A). The “SOB score” representing strand bias^16^ was higher in the FFPE artifacts (Fig. 3B). A score closer to 1 indicates higher bias, whereas a score closer to 0 represents lower bias. In terms of the type of single nucleotide variants (SNVs), the variants called in the FFPE samples were predominantly C>T and G>A. The number of C:G>T:A substitutions was higher in the FFPE-artifacts than in the true variants in breast and lung cancers (Fig. 4A, B), where the majority of detected variants were FFPE-artifacts (Fig. 2). Interestingly the most dominant type of SNV in fibrosarcoma was G>T substitutions due to guanine oxidation^17^. These data demonstrate the adequacy of the compiled WES dataset we used to represent diverse types of FFPE-induced artifacts. Moreover, we confirmed that the FFPE-artifacts identified in this study have typical properties previously observed^2,4,6,16^.

### Existing tools for removal of FFPE-induced artifacts are not robust

The MuTect filter called “FilterByOrientationBias” can be applied to exclude artifactual mutations from somatic variant calls. It was designed to remove artifacts induced by guanine oxidation and cytosine deamination, which result in G:C>T:A transversion and C:G>T:A transition, respectively. We wanted to check if FilterByOrientationBias could remove FFPE-artifacts. Although the filter retained true variants with a sensitivity of 0.969, it removed only 40.7% of the artifacts (11,204 of 27,510) (“All (public WES dataset)” for MuTect filter in Table 1). Consequently, only 2.4% of the predicted variants were actually true variants, and the rest were artifacts that should have been filtered out. Regardless of the cancer type tested, the filter did not work effectively. Specifically, it failed to remove any FFPE-artifacts from the liver and colon cancer data. In breast cancers, the precision, i.e., the fraction of true somatic variants among predicted somatic variants, was 0.013 (Table 1). This result suggests that the filter is not feasible for clinical applications.

**Table 1:** Assessment of the ability of MuTect filter, FFPolish, SOBDetector, and DEEPOMICS FFPE.

		True positives (True variants)	True negatives (True artifacts)	False positives	False negatives	Specificity	Sensitivity	Precision	F1-score	Accuracy
MuTect filter	All (public WES dataset)	401	11,204	16,306	13	0.407	0.969	0.024	0.047	0.416
	Fibrosarcoma	43	0	41	0	0.000	1.000	0.512	0.677	0.512
	Lung cancer	138	124	917	2	0.119	0.986	0.131	0.231	0.222
	Liver cancer	7	0	97	0	0.000	1.000	0.067	0.126	0.067
	Colon cancer	17	0	53	0	0.000	1.000	0.243	0.391	0.243
	Breast cancer	196	11,080	15,198	11	0.422	0.947	0.013	0.025	0.426
	G:C>T:A	47	0	1062	0	0.000	1.000	0.042	0.081	0.042
	C:G>T:A	142	11,204	11,603	13	0.491	0.916	0.012	0.024	0.494
	Low MAF variants	4	110	1360	0	0.075	1.000	0.003	0.006	0.077
	A549 cells (WES)	739	0	51	0	0.000	1.000	0.935	0.967	0.935
	A549 cells (Targeted panel seq)	15	0	6	0	0.000	1.000	0.714	0.833	0.714
FFPolish	All (public WES dataset)	282	25,607	1903	132	0.931	0.681	0.129	0.217	0.927
	Fibrosarcoma	32	33	8	11	0.805	0.744	0.800	0.771	0.774
	Lung cancer	104	949	92	36	0.912	0.743	0.531	0.619	0.892
	Liver cancer	0	87	10	7	0.897	0.000	0.000	0.000	0.837
	Colon cancer	13	42	11	4	0.792	0.765	0.542	0.634	0.786
	Breast cancer	133	24,496	1782	74	0.932	0.643	0.069	0.125	0.930
	G:C>T:A	41	1028	34	6	0.968	0.872	0.547	0.672	0.964
	C:G>T:A	120	21,012	1795	35	0.921	0.774	0.063	0.116	0.920
	Low MAF variants	0	1453	17	4	0.988	0.000	0.000	0.000	0.986
	A549 cells (WES)	648	47	4	91	0.922	0.877	0.994	0.932	0.880
	A549 cells (Targeted panel seq)	13	6	0	2	1.000	0.867	1.000	0.929	0.905
SOBDetector	All (public WES dataset)	352	19,852	7658	62	0.722	0.850	0.044	0.084	0.724
	Fibrosarcoma	38	16	25	5	0.390	0.884	0.603	0.717	0.643
	Lung cancer	125	585	456	15	0.562	0.893	0.215	0.347	0.601
	Liver cancer	2	62	35	5	0.639	0.286	0.054	0.091	0.615
	Colon cancer	15	30	23	2	0.566	0.882	0.395	0.545	0.643
	Breast cancer	172	19,159	7119	35	0.729	0.831	0.024	0.046	0.730
	G:C>T:A	47	848	214	0	0.798	1.000	0.180	0.305	0.807
	C:G>T:A	143	15,745	7062	12	0.690	0.923	0.020	0.039	0.692
	Low MAF variants	3	768	702	1	0.522	0.750	0.004	0.008	0.523
	A549 cells (WES)	698	24	27	41	0.471	0.945	0.963	0.954	0.914
	A549 cells (Targeted panel seq)	13	5	1	2	0.833	0.867	0.929	0.897	0.857
DEEPOMICS FFPE	All (public WES dataset)	293	27,408	102	121	0.996	0.708	0.742	0.724	0.992
	Fibrosarcoma	37	35	6	6	0.854	0.860	0.860	0.860	0.857
	Lung cancer	110	996	45	30	0.957	0.786	0.710	0.746	0.936
	Liver cancer	5	91	6	2	0.938	0.714	0.455	0.556	0.923
	Colon cancer	16	49	4	1	0.925	0.941	0.80	0.865	0.929
	Breast cancer	125	26,237	41	82	0.998	0.604	0.753	0.670	0.995
	G:C>T:A	38	1047	15	9	0.986	0.809	0.717	0.760	0.978
	C:G>T:A	89	22,786	21	66	0.999	0.574	0.809	0.672	0.996
	Low MAF variants	1	1462	8	3	0.995	0.250	0.111	0.154	0.993
	A549 cells (WES)	711	33	18	28	0.647	0.962	0.975	0.969	0.942
	A549 cells (Targeted panel seq)	15	6	0	0	1.000	1.000	1.000	1.000	1.000

Another option is FFPolish, which removes FFPE-artifact calls based on the characteristics of the artifacts, such as allele frequency, size of insert fragment, and strand bias of the artifacts^18^. While FFPolish had higher specificity values of 0.931 (“All (public WES dataset)” for FFPolish in Table 1), it did not work well on the breast cancer samples with a precision of 0.063 (Table 1).

### DEEPOMICS FFPE, a deep neural network model to classify true variants from artifactual mutations

As previously described, the datasets generated from different groups reflect diverse DNA and sequencing data qualities. Coping well with that diverse circumstances should be paramount to differentiate between the artifacts and the variants. Thus, we developed DEEPOMICS FFPE, a classifier that employs deep neural networks.

FFPE-artifacts have some distinguishable properties, such as low MAF and strand bias. We attempted to excavate other properties that can be used to train the deep neural networks and found that some properties show significant differences between true variants and FFPE-artifacts, for example, the position of mutation and cosine similarity between strand-orientation bias for reference allele reads and for alternate allele reads (Supplementary Fig. S1). In addition, we included some categorical properties, such as whether a given variant is SNV or insertion or deletion (all predictor variables and descriptions of them are listed in Supplementary Table S2). We determined 41 predictor variables using the output of the MuTect2 caller and established deep neural networks composed of 41 input nodes and 3 hidden layers with binary cross entropy as a loss function. The output of the networks is a probability of being a true variant for each called variant. We used 70% of the compiled WES dataset for training and the rest for validation.

In the validation set, we observed improved precision (0.742) after using DEEPOMICS FFPE (“All (public WES dataset)” for DEEPOMICS FFPE in Table 1). Specifically, 99.6% of the FFPE-artifacts (27,408 of 27,510) were successfully eliminated while retaining 70.8% (293 of 414) of the true variants (“All (public WES dataset)” for DEEPOMICS FFPE in Table 1). The datasets used to train the model predominantly were composed of data generated from breast cancers, suggesting that the model was effective on specific samples similar to breast cancer samples used for training. The performance of the model was assessed within each cancer type. In breast cancer, the artifacts were removed almost perfectly with a specificity of 0.998 (26,237 of 26,278 FFPE-artifacts removed); however, 82 of 207 true variants were misclassified with a resulting sensitivity of 0.604 (“Breast cancer” for DEEPOMICS FFPE in Table 1). Similar results were observed in the liver cancer samples (specificity: 0.938, and sensitivity: 0.714). The sensitivity and specificity were balanced in the other cancer samples. These results show that DEEPOMICS FFPE is superior to MuTect filter and FFPolish in terms of F1-score and specificity.

**Figure 5:**
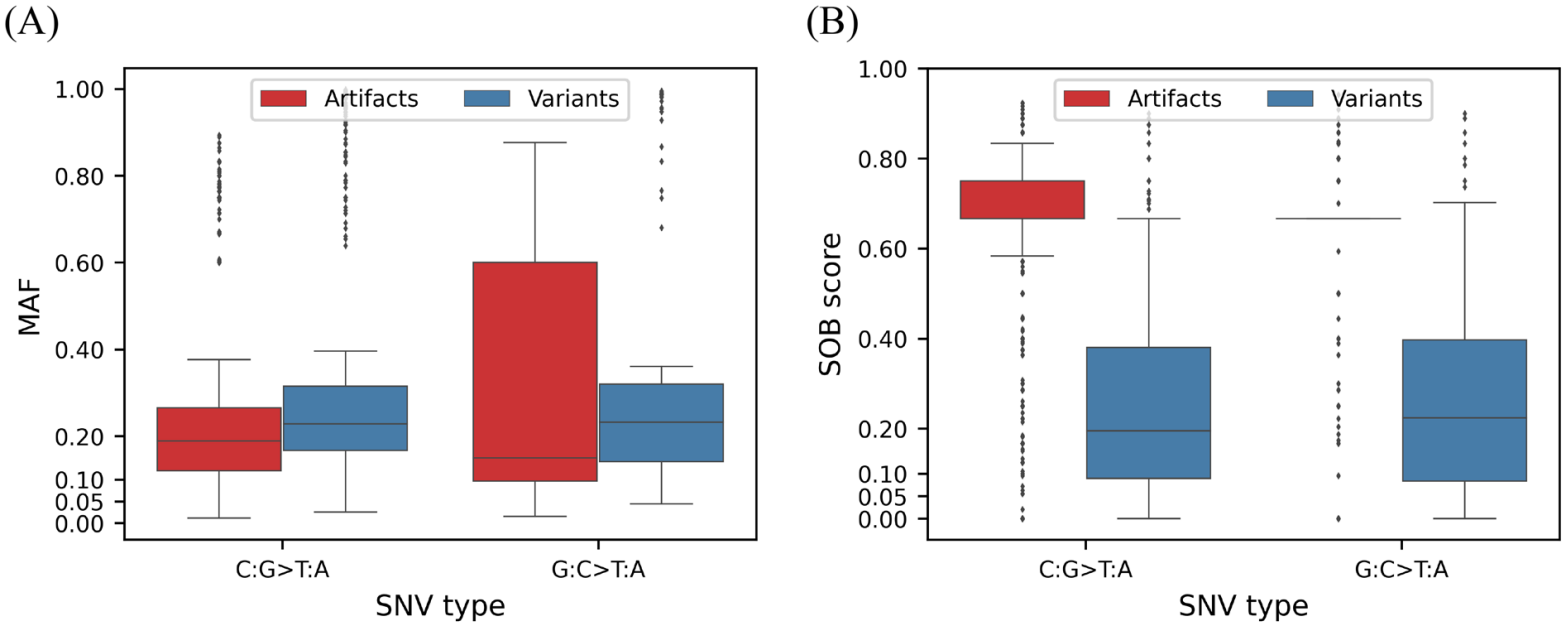
G:C>T:A artifacts have low MAF and strand bias as C:G>T:A artifacts. MAF (**A**) and SOB scores (**B**) were plotted for FFPE-artifacts in red and for true variants in blue. X-axis indicates SNV types. (****p-value < 0.0001, ***p-value < 0.001, *p-value < 0.05, one-tailed Mann–Whitney *U*-test).

Intriguingly, G:C>T:A artifacts were also removed with a specificity of 0.986 (1047 of 1062 removed) by DEEPOMICS FFPE (“G:C>T:A” for DEEPOMICS FFPE in Table 1). Notably, FFPolish also removed most of the G:C>T:A artifacts with a specificity of 0.968 (1028 of 1062) (“G:C>T:A” for FFPolish in Table 1). A plausible explanation of how DEEPOMICS FFPE and FFPolish distinguish G>T artifacts is that G>T artifacts shared some characteristics with C>T artifacts. The MAF of G:C>T:A artifacts (median value: 0.15) was significantly less than that of G:C>T:A variants (median value: 0.227; one-tailed Mann–Whitney *U*-test, *p*-value < 0.005) (Fig. 5A right), as observed between C:G>T:A artifacts and variants (Fig. 5A left). Similarly, the SOB score showed that G:C>T:A artifacts were strand biased compared with their counterpart variants similar to C:G>T:A artifacts (median: 0.66) (Fig. 5B).

### DEEPOMICS FFPE can be used to identify true somatic variants with low allele frequencies

Artifacts from FFPE tissues generally have low allele frequencies (Fig. 2A)^2,4^. Thus, discrimination of true somatic variants with low MAFs from artifacts is important to identify subclonal mutations or driver mutations in samples with a low tumor cellularity, such as pancreatic cancer^19,20^. Existing tools perform poorly in this aspect. We tested whether DEEPOMICS FFPE can classify variants with 1%–5% MAFs (“Low MAF variants” for DEEPOMICS FFPE in Table 1). DEEPOMIC FFPE removed artifacts with a specificity of 0.995 and preserved true variants with a sensitivity of 0.25. By contrast, MuTect filter removed only 7.5% of the artifacts (“Low MAF variants” for MuTect filter in Table 1). Consequently, 0.111% of the true variants remained among the predicted variants. Similar to DEEPOMICS FFPE, FFPolish successfully eliminated 98.8% of the FFPE-artifacts (“Low MAF variants” for FFPolish in Table 1). However, it misclassified all true variants as artifacts.

### Evaluation of DEEPOMICS FFPE using well-defined datasets

We considered the variant calls detected in the FFPE-only samples as FFPE-artifacts. However, we cannot rule out the possibility that some of the FFPE-only artifacts are true subclonal variants not detected in the matched FF sample due to regional genetic heterogeneity. Thus, we used FF and FFPE samples with the same genetic make-up. Additionally, we wanted to evaluate DEEPOMICS FFPE on a new dataset. Thus, we used the A549 (ATCC number: CCL-185) cell line isolated from a patient with lung cancer. We prepared the FFPE block and FF sample from the same batch of the cultured A549 cells. DNAs extracted from matched samples were subjected to WES and targeted capture sequencing with a custom cancer panel. After WES, we observed that the insert fragments from the FFPE block (median length: 147 bp) were shorter than those from the matched FF (median length: 221 bp) (Supplementary Fig. S2) similar to data from clinical samples (Fig. 1B–F). This result indicated that formaldehyde-induced fragmentation occurred, with some artifactual mutations in the FFPE samples. In WES, 790 variants were called from FFPE, of which 51 were FFPE-artifacts. DEEPOMICS FFPE removed 64.7% of the FFPE-artifacts (33 of 51); consequently, 97.5% (711 of 729) of the predicted variants were true variants (“A549 cells (WES)” for DEEPOMICS FFPE in Table 1). To evaluate the performance of DEEPOMICS FFPE and other tools in classifying variants called from high-depth sequencing data, we carried out targeted sequencing with customized cancer panels for the cultured cells. Twenty-one variants were called from FFPE, of which six variants were FFPE-artifacts. DEEPOMICS FFPE perfectly classified all true variants and FFPE-artifacts (“A549 cells (Targeted panel seq)” for DEEPOMICS FFPE in Table 1). However, FFPolish and SOBDetector misclassified two true variants as FFPE-artifacts. MuTect filter misclassified all six FFPE-artifacts as true variants.

To test the performance of the tools on true variants that were previously validated, we performed the targeted capture sequencing of DNA extracted from a commercial reference standard for FFPE (catalog number: HD200, Horizon). Following the manufacturer’s manual, 11 driver mutations in *BRAF*, *KIT*, *EGFR*, *KRAS*, *NRAS*, and *PIK3CA* were validated (Table 2). In the somatic variant calling workflow used in this study, 10 of the 11 variants were called by MuTect2. *EGFR* T790M was not called presumably because of its low allele frequency (expected allele frequency: 1%). As expected, MuTect filter and SOBDetector showed high sensitivities of 1 (10 out of 10) and 0.9 (9 out of 10), respectively. Meanwhile, DEEPOMICS FFPE classified seven driver mutations as true variants with a sensitivity of 0.7 (7 out of 10) and misclassified two driver mutations (*EGFR* ΔE746-A750, *KRAS* G12D, *PIK3CA* E545K). FFPolish correctly classified six true variants. Taken together, these data confirmed that DEEPOMICS FFPE can effectively discriminate between FFPE-artifacts and true variants.

**Table 2:** List of validated variants in the standard material and the results of the inference of the indicated tools.

Gene	Mutation	SNV	Chrom.^†^	Position	Allele frequency (expected)	Allele frequency (observed)	Depth	Num. of reads supporting alt. allele	MuTect filter	FFPolish	SOB detector	DEEPOMICS FFPE
*BRAF*	V600E	T>A	chr7	140453136	0.105	0.115	388	45	O	O	O	O
*KIT*	D816V	A>T	chr4	55599321	0.1	0.573	623	357	O	O	O	O
*EGFR*	delE746-A750	-	chr7	55242463	0.02	0.012	3163	36	O	X	X	X
*EGFR*	L858R	T>G	chr7	55259515	0.03	0.033	2710	101	O	X	O	X
EGFR*	T790M	C>T	chr7	55249071	0.01	0.009	2528	25	N/D^‡^	N/D	N/D	N/D
*EGFR*	G719S	G>A	chr7	55241707	0.245	0.241	1074	264	O	O	O	O
*KRAS*	G13D	G>A	chr12	25398281	0.15	0.127	1827	236	O	O	O	O
*KRAS*	G12D	G>A	chr12	25398284	0.06	0.098	1824	182	O	X	O	O
*NRAS*	Q61K	C>A	chr1	115256530	0.125	0.143	1784	258	O	O	O	O
*PIK3CA*	H1047R	A>G	chr3	178952085	0.175	0.143	171	24	O	X	O	O
*PIK3CA*	E545K	G>A	chr3	178936091	0.09	0.556	201	112	O	O	O	X

*In case of *EGFR* T790M, the observed allele frequency, depth, and number of reads supporting alternate allele were obtained from the bam file with IGV genome browser (https://igv.org/), because the variant was not called by MuTect2.

^†,‡^Stands for chromosome and not detected, respectively.

## Discussion

FFPE allows the archival of clinical samples at room temperature for several decades without compromising histomorphology. However, FFPE specimens contain artifactual mutations. Thus, removal of such artifact is important to improve variant calling for precision medicine.

DEEPOMICS FFPE is effective in removing FFPE artifacts, although some true variants were inadvertently filtered out. This might not align with the goals of researchers seeking more true variants. To address this, the cutoff value, which distinguishes between artifacts and true variants, could be fine-tuned. By default, DEEPOMICS FFPE employs a value of 0.5. We realized that adjusting the value to 0.425 maximizes the F1-score. Implementing this change increases sensitivity from 0.708 to 0.717. However, it is important to note that this adjustment might increase the number of false positives.

A previous study showed that the sensitivity and precision of variant calling can be improved by introducing “at least 2 callers”^21^. In the study, the parameters of four different callers (MuTect2, VarScan2^22^, Strelka2^23^, and Shimmer^24^) were independently optimized to maximize the overlap between the FF and its FFPE counterpart. Considering the variants called by at least two optimized callers as true variants improved the F1-score to 0.829 for the same dataset used for optimization. However, F1-scores of 0.0647–0.87167 on the different datasets imply that the strategy probably works well only on specific datasets. When we tested it with the same parameters used in the previous study on our lung cancer dataset, several artifacts were classified as true variants (Supplementary Table S3). As a result, we observed the F1-score of 0.13, which is not robust. We hypothesized that AI-based algorithms rather than rule-based approaches such as the “at least 2 caller” strategy can cope effectively with various DNA and sequencing data qualities affected by FFPE. Consistent with this hypothesis, we showed that FFPolish, which employs the machine learning algorithm logistic regression, can make better predictions than rule-based approaches.

Even if an AI algorithm is outstanding, a decision should be explained before its performance can be trusted. We attempted to identify relevant features to assess whether the decision of DEEPOMICS FFPE is reliable. We found that MAF, SOB score, and SNV type are important features using SHapley Additive exPlanations, which can identify features that are relevant for a machine learning algorithm to make predictions^25^. This result indicates that the prediction of DEEPOMICS FFPE is reasonable because the relevance of the features was expected as previously shown (Figs. 3 and 4). To gain deep insights into the contribution of the features for the prediction, we extracted the outputs of the second hidden layer and projected them into two-dimensional space by using UMAP (Uniform Manifold Approximation and Projection for Dimension Reduction^26^). First, we superimposed how confidently DEEPOMICS FFPE classified a given variant as a true variant on the space (Supplementary Fig. S3). In the figure, darker red indicates that DEEPOMICS FFPE confidently classifies a given variant as a somatic variant, whereas darker blue represents it confidently classifies a given variant as a FFPE-artifact. Interestingly, variants classified as somatic by DEEPOMICS FFPE with high confidence are localized together (a rectangle in Supplementary Fig. S3B and S3D). In the case of true FFPE-artifacts in the rectangle, DEEPOMICS FFPE does not seem confident that the artifacts are real (Supplementary Fig. S3A and S3C). To understand the contribution of SOB score to the confidence of DEEPOMICS FFPE, we superimposed the SOB score as the confidence of DEEPOMICS FFPE (Supplementary Fig. S4). The somatic variants confidently predicted were less strand biased (rectangle in Supplementary Fig. S4B and S4D), whereas the variants classified as artifacts were strand biased (rectangle in Supplementary Fig. S4A and S4C). This result indicated that the SOB score provided discriminating power to variant classification in the area where DEEPOMICS FFPE confidently predicted true somatic variants. We also checked whether MAF contributes discriminating power by plotting MAF on the space (Supplementary Fig. S5). Unlike SOB score, MAF did not provide strong discriminating power in the area. However, MAF allowed DEEPOMICS FFPE to exclude some artifacts with high allele frequency by making them locate in distinct areas. We analyzed the effect of the combination of MAF and SOB score on making predictions. As shown in Supplementary Fig. S5, MAF helped DEEPOMICS FFPE discriminate FFPE-artifacts with high allele frequencies (Supplementary Fig. S6). Interestingly, the combination of MAF and SOB score helped discriminate true FFPE-artifacts with MAF < 0.5 from true somatic variants. We tested various combinations between the features listed in the Supplementary Table S2 (data not shown) and concluded that the process by which DEEPOMICS FFPE makes predictions can be explained and expectable.

The mutational signatures of cancer tissues can provide an insight into the mutation processes during cancer development. For example, mutation signatures SBS2 and SBS13 are associated with the activation of APOBEC (Apolipoprotein B mRNA editing enzyme, catalytic polypeptide), a major driver of subclonal evolution of the cancer genome^27,28^. Given that APOBEC3B catalyzes cytosine deamination similar to FFPE, FFPE-artifacts may be misinterpreted as APOBEC mutation signature, while true mutations caused by APOBEC3B could be misclassified as FFPE-artifacts by DEEPOMICS FFPE. In this context, we attempted to characterize the single base substitution signature of WES data from FF, FFPE, and the FFPE after applying DEEPOMICS FFPE for breast cancer samples. For this, we inevitably had to use all mutation calls, including the calls used for model training, because the mutation signature analysis requires as many mutations as possible for its comprehensiveness. Results showed that all FFPE samples for breast cancers, except for “Breast cancer-9,” showed SBS2 related to hyper-active APOBEC; however, eight of the FFPE samples were not observed in the matched FF samples (Supplementary Fig. S7). This result indicates that the FFPE-induced artifactual mutations could mislead the given cancer to have an APOBEC-positive signature. This observation was not reported in the previous study^4^. After applying DEEPOMICS FFPE (Supplementary Fig. S7C), the contribution of SBS2 in FFPE from “Breast cancer-13” was consistent (Supplementary Fig. S7C) with the observation in the matched FF samples (Supplementary Fig. S7A). This result implies that “Breast cancer-13” has an APOBEC-positive signature and that DEEPOMICS FFPE helped refine patient-specific mutational signature by removing FFPE-artifacts although SBS2 signature that does not seem to be real was detected in “Breast cancer-9” by applying DEEPOMICS FFPE. Three FF samples (Breast cancer-1, -2, and -7) had SBS13, but they were not detected in the matched FFPE samples (Supplementary Fig. S7A and S7B). DEEPOMICS FFPE could salvage two of them while misclassifying one non-SBS13 cases as SBS13 (Supplementary Fig. S7C). Overall, if we were to classify cases with either SBS2 or SBS13 as APOBEC activated, 5 of 13 FF cases (Breast cancer-1, -2, -7, -10, and -13), 12 of 13 FFPE cases (all samples except for Breast cancer-9), and 4 DEEPOMICS FFPE cases (Breast cancer-2, -7, -9, and -13) would be classified as APOBEC activated. SBS1 and SBS30 were exclusively observed in the FFPE samples, which was consistent with the previous finding that FFPE-signature is similar to SBS1 and SBS30^4^. Impressively, all SBS30 signatures observed in FFPE disappeared after applying DEEPOMICS FFPE, whereas “Breast cancer-2” still showed SBS1 even after DEEPOMICS FFPE (Supplementary Fig. S7C). Surprisingly, SBS11, which is associated with previous temozolomide treatment, was observed in all FFPE samples^29^. This result was not reported in the previous study^4^. Considering that that the signature catalog was not observed in the matched FF sample, it should be related to FFPE-artifacts. Eventually, the SBS11 detected in FFPE was removed by DEEPOMICS FFPE except “Breast cancer-6”. SBS15 signatures were detected in all the FF samples but were absent in the FFPE samples. Even after applying DEEPOMICS FFPE, the signature remained undetected. According to COSMIC documentation, SBS15 exhibits predominant GCG>GTN alterations. It is possible that DEEPOMICS FFPE misclassifies cases where the true alterations are GCN>GTN, although the exact cause is yet unknown. Exploring whether the sequence context contributes to artifact removal would be interesting for further study. In summary, this study demonstrates that DEEPOMICS FFPE has the potential to refine signatures associated with certain etiologies.

We have shown the performance of our newly developed tool DEEPOMICS FFPE trained on datasets publicly available. We observed varying numbers of FFPE artifacts and characteristics among the datasets. The origin of these differences remains unclear, whether they come from sample-specific entities or the chemistry used during dataset generation, although it is not necessarily mutually exclusive. Consequently, the datasets may reflect limited specific entities or chemistry. It is conceivable that DEEPOMICS FFPE has only learned a restricted spectrum of information represented in these datasets.

To ensure the universal applicability of DEEPOMICS FFPE, we sequenced DNA from FFPE-blocks of cultured cells, confirming its effectiveness in removing artifacts from ‘unseen’ samples. Nevertheless, it is imperative to validate DEEPOMICS FFPE on datasets derived from diverse entities and generated using varying chemistry. Datasets from high-TMB cancers, such as melanoma, or defective DNA repair signatures should be worth generating because these samples contain several mutations with various allele frequencies. We hope we can collaborate with academia that can generate these datasets to improve personalized medicine.

## Methods

### WES datasets

We obtained WES datasets for five types of cancer, including 24 FFPE samples and 24 matched FF samples (Supplementary Table S1). Data of one fibrosarcoma and two lung cancers were downloaded from Sequence Read Archive (SRA) with the accession number PRJNA301548. Data of four liver cancers and four colon cancers were downloaded from European Genome-Phenome Archive (EGA) with the accession number EGAS00001002631. Although the liver and colon cancer tissues were obtained from a single patient each, we treated them as separate samples for data augmentation. For breast cancers, we downloaded 13 matched FFPE and FF datasets from SRA with the accession number SRP044740. In the case of breast cancers, some technical replicates (Supplementary Table S1) were used as separate samples for data augmentation.

### WES workflow

The fastq files that were downloaded underwent quality control and adapter trimming. We aligned the sequences to the human reference genome (hg19 assembly) and removed potential PCR duplicates using BWA and Picard. Prior to variant calling, the base calls were recalibrated using the GATK toolkit. MuTect2, which is widely used and known for its stability and relative accuracy^12,14,15^, was used as the somatic variant caller. The variants were annotated using SnpEff, a genetic variant annotation tool^30^. We used the variants that fulfilled a PASS filter.

To visualize the distributions of the length of insert fragments, we obtained concordantly mapped reads that met the following criteria; properly mapped, not duplicated, not secondary, not supplementary, and mapping quality greater than 20. Then the length of the fragments was obtained using pysam, a python module for reading BAM file. The SOB scores were calculated as previously described^16^, and MAF from variant call format (VCF) was visualized with matplotlib, a python library for visualization^31^.

### Development of DEEPOMICS FFPE and evaluation of DEEPOMICS FFPE, MuTect filter, and FFPolish

To avoid any preoccupation and evaluate DEEPOMICS FFPE and existing tools (MuTect filter, FFPolish, and SOBDetector) fairly, variant calls from every single vcf file were divided into a random train and a validation dataset at a ratio of 7 (train dataset):3 (validation dataset). For this, we used the train_test_split function of scikit-learn (https://scikit-learn.org) with the “random_state=42” option^32^. Although we used technical replicates as independent samples for model training, we excluded the variants overlapped with the variants used for model training when we evaluated the tools.

The features extracted for DEEPOMICS FFPE and their descriptions are listed in supplementary Table S2. DEEPOMICS FFPE consists of three linear layers with 41, 32, and 16 nodes. The first two layers each have a rectified linear unit (ReLU) activation function^33^. Two batch normalization layers were added between hidden layers to improve performance^34^. Softmax function was used for the output that represents a probability of being a true variant for each called variant. Binary cross entropy and Adam algorithm were used to compute loss and update model parameters, respectively^35^. The model was implemented with PyTorch (version 1.11.0)^36^.

The same datasets were used to evaluate DEEPOMICS FFPE, MuTect filter^13^, SOBDetector^16^, and FFPolish^18^. Accuracy, specificity, sensitivity (also known as recall), precision, and F1-score were calculated as follows:

$${\text{Accuracy }} = \, \left( {{\text{TP }} + {\text{ TN}}} \right)/\left( {{\text{TP }} + {\text{ TN }} + {\text{ FP }} + {\text{ FN}}} \right)$$

$${\text{Specificity }} = {\text{ TN}}/\left( {{\text{TN }} + {\text{ FP}}} \right)$$

$${\text{Sensitivity }} = {\text{ TP}}/\left( {{\text{TP }} + {\text{ FN}}} \right)$$

$${\text{Precision }} = {\text{ TP}}/\left( {{\text{FP }} + {\text{ TP}}} \right)$$

where TP, TN, FP, and FN are true positives, true negatives, false positives, and false negatives, respectively.

$${\text{F1 - score }} = { 2}/\left( {{\text{sensitivity}}^{{ - {1}}} + {\text{ precision}}^{{ - {1}}} } \right)$$

### Preparing FFPE block for A549 cells

A549 (MERCK) cells were grown on RPMI 1640 medium supplemented with 10% fetal bovine serum (Cytiva) and 1% penicillin/streptomycin (Gibco) in a roller bottle (Jet Bio-Filtration). The cells were washed with Dulbecco’s phosphate buffered saline (Gibco) and then harvested with Accutase (Sigma). Prior to centrifugation, the cells were divided into two tubes for FFPE and FF samples. After centrifugation, the cells in the tube for FF were stored in liquid nitrogen until ready to use. For the FFPE samples, cells at 1 × 10^8^ were subjected to fixation with neutral buffered 4% paraformaldehyde (Cellnest) at 4 °C for 24 h. After centrifugation at 250 × g for 5 min, the pellets were resuspended with 4% low-melting-point agarose (Invitrogen) and solidified on ice for 3 min. The samples were stored in 4% paraformaldehyde (Cellnest) for 24 h and then embedded with melted paraffin in an embedding cassette. The FFPE block was stored at room temperature for about 2 months before DNA extraction.

### Sequencing for A549 cells and the standard reference material

The GeneRead kit (Qiagen) was used to extract DNA from the FFPE block for A549 cells and the standard material (HD200, Horizon). Although there is a step to treat UDG enzyme to remove dU from DNA molecules in accordance with the manufacturer’s instructions, we did not treat it. To obtain sufficient artifactual mutations, we added the same volume of nuclease-free water instead of the enzyme in the step. Exome was captured and libraries were prepared using the SureSelect V5 enrichment capture kit (Agilent). The libraries for targeted sequencing were prepared using our customized cancer panel (gene number of 359; panel size of 1.66 Mb) produced by Agilent in accordance with the manufacturer’s instructions. The libraries were sequenced using the NovaSeq 6000 system (Illumina).

### Characterization of mutational signatures

To characterize the mutational signatures of breast cancers, we used Mutalisk, a web-based somatic mutation analysis toolkit with default options^37^. Considering that the breast cancer datasets include multiple replicates, we used the union set of variant calls for each breast cancer sample. In addition, mutational signature analysis requires as many variant calls as possible. Hence, we used all variant calls, including the variants used for training.

### Supplementary Information

Supplementary Figures.

Supplementary Table S1.

Supplementary Table S2.

Supplementary Table S3.

## Data Availability

The datasets generated during this study are available in SRA under accession number (PRJNA991305). Public sequence datasets used in the study are available in SRA under accession number (PRJNA301548 and SRP044740) and in EGA under accession number (EGAS00001002631). Web-based DEEPOMICS FFPE is available on the web (http://deepomics.co.kr/ffpe).
