# Supplementary material for: DEEPOMICS FFPE, a deep neural network model, identifies DNA sequencing artifacts from formalin fixed paraffin embedded tissue with high accuracy
Source: Sci Rep. 2024 Jan 31;14:2559. doi: 10.1038/s41598-024-53167-0 (PMC10831091; doi:10.1038/s41598-024-53167-0)
Supplement: Supplementary file 1 — Supplementary Figures. [file 41598_2024_53167_MOESM1_ESM.docx]

DEEPOMICS FFPE, a deep neural network model, identifies DNA sequencing artifacts from formalin fixed paraffin embedded tissue with high accuracy

Dong-hyuk Heo^1^, Inyoung Kim^1^, Heejae Seo^1^, Seong-Gwang Kim^1^, Minji Kim^1^, Jiin Park^1^, Hongsil Park^1^, Seungmo Kang^1^, Juhee Kim^1^, Soonmyung Paik^1^, Seong-Eui Hong^1,^*

^1^Theragen Bio Co., Ltd., Seongnam, Gyeonggi-do, 13488, Republic of Korea

*To whom correspondence should be addressed

Contact: seongeui.hong@theragenbio.com

**Supplementary Information**

(B)


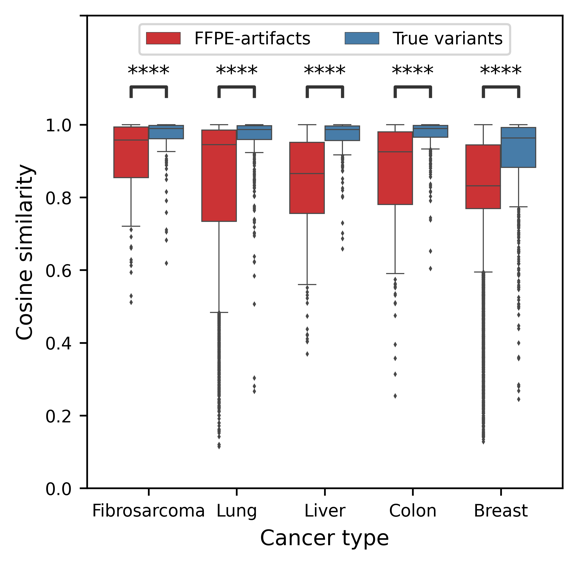

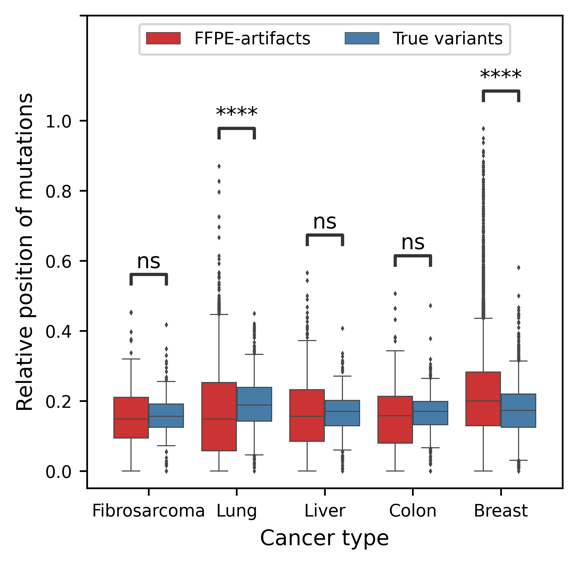


(A)

**Supplementary Fig. S1 Properties of FFPE-artifacts different from true variants**

Relative position of mutations (A) and cosine similarity of strand-orientation bias between reference allele and alternate allele (B) were plotted for FFPE-artifacts in red and for true variants in blue. X-axis indicates the cancer samples used in this study. **** denotes statistical significance (p-value < 0.0001, Mann-Whitney *U*-test) and ns means the difference is not significant.


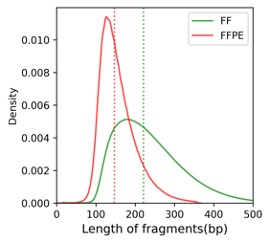


**Supplementary Fig. S2 The length of insert fragments from WES for A549 cells**

The distribution of the length of insert fragment from FF (green line) and FFPE (red line) were plotted. The vertical dotted lines indicate the median values for the length of insert from FF (green line) and FFPE (red line).


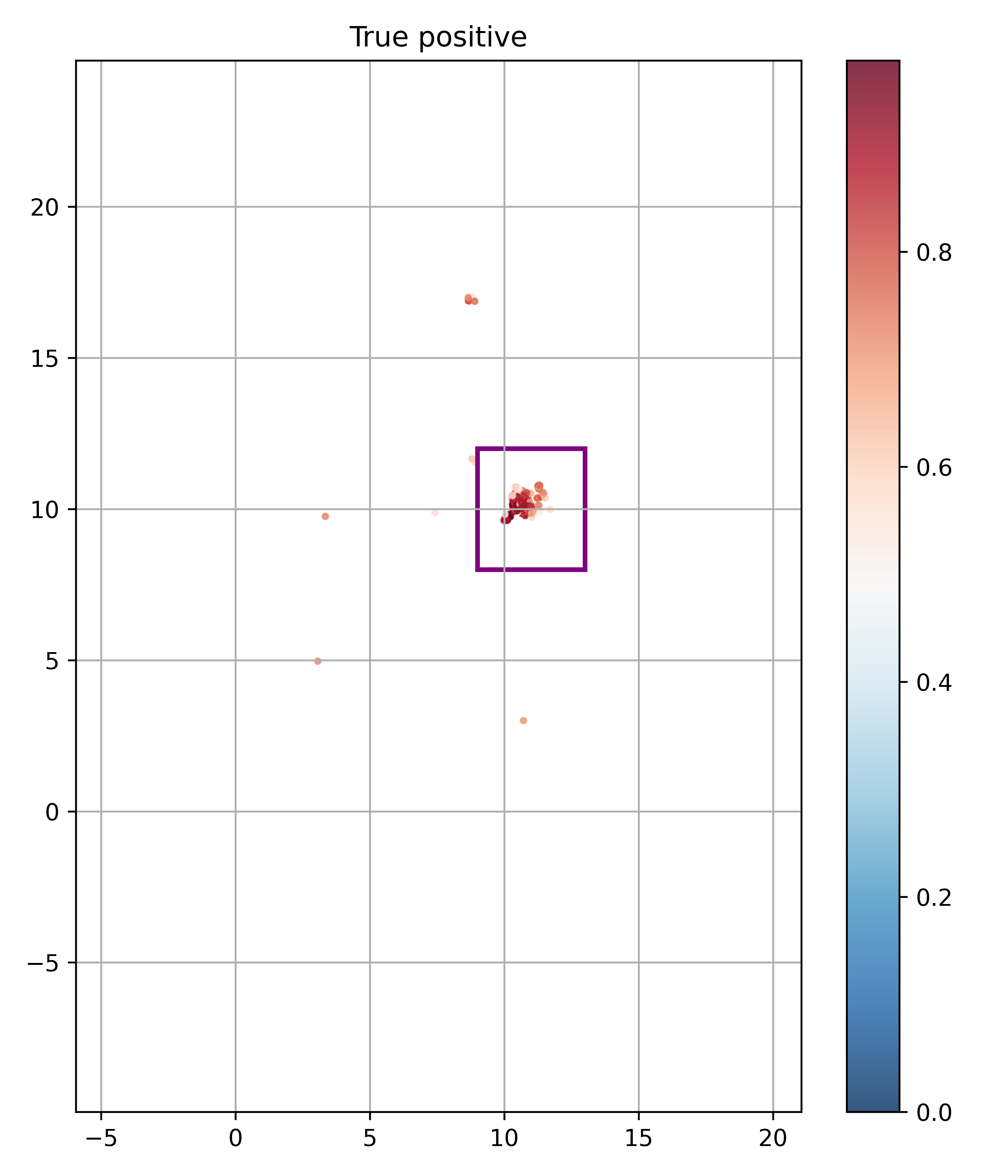

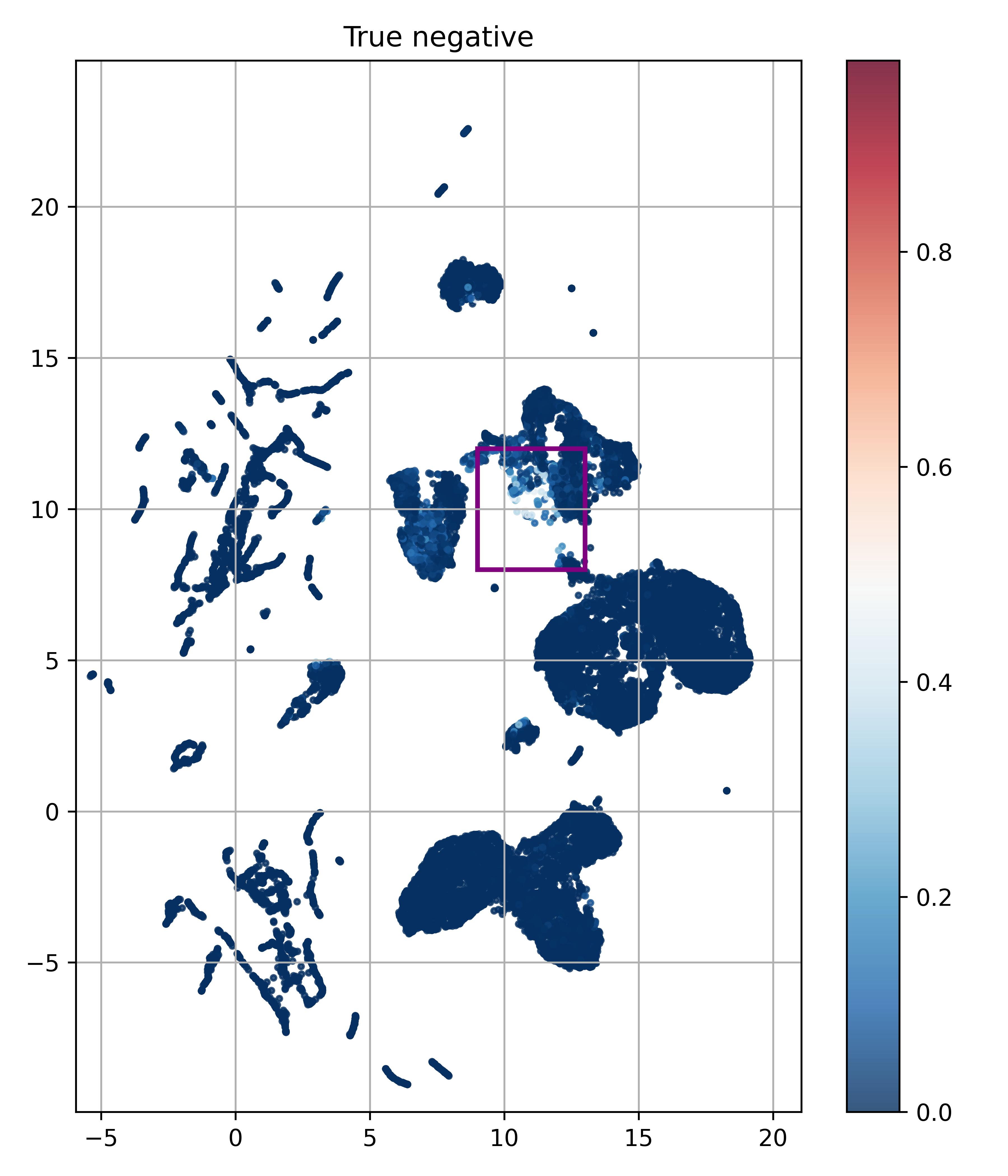

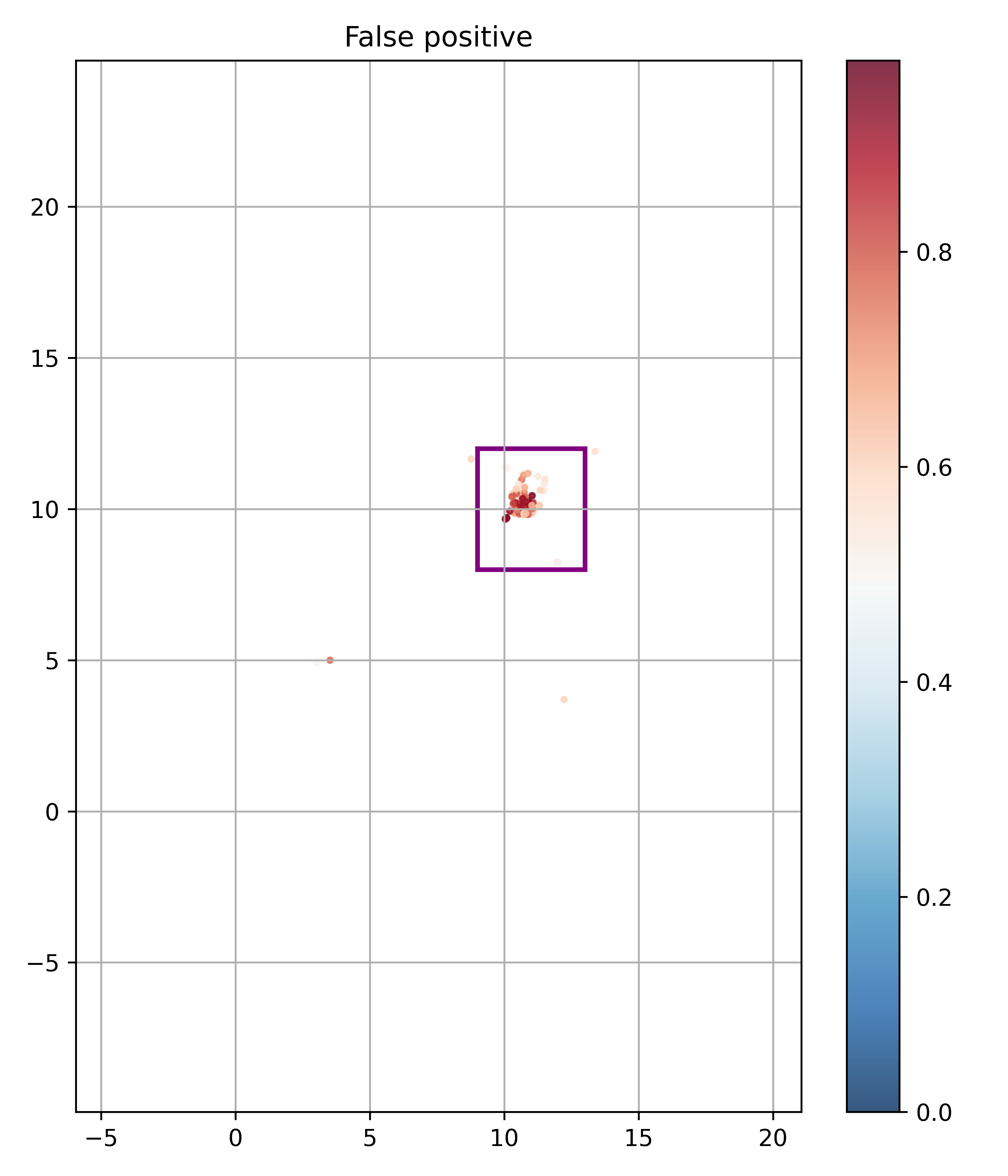

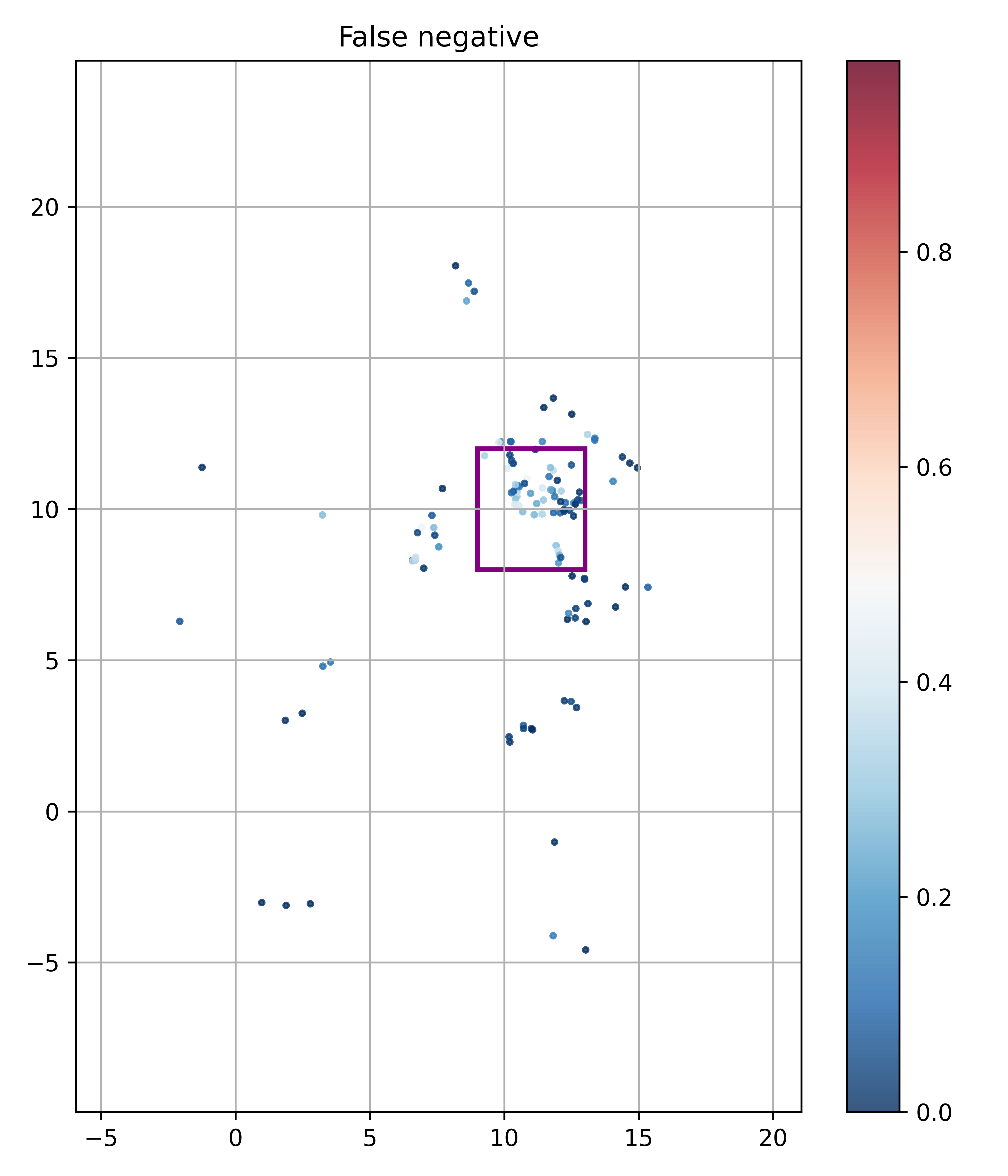


(D)

(C)

(B)

(A)

**Supplementary Fig. S3** UMAPs represent the outputs of the second hidden layer of DEEPOMICS FFPE for true FFPE-artifacts (A), false variants (B), false FFPE-artifacts (C), and true variants (D). The color scale represents how confidently DEEPOMICS FFPE is sure of a true variant. The purple rectangles indicate the areas where DEEPOMICS FFPE is mostly confident.


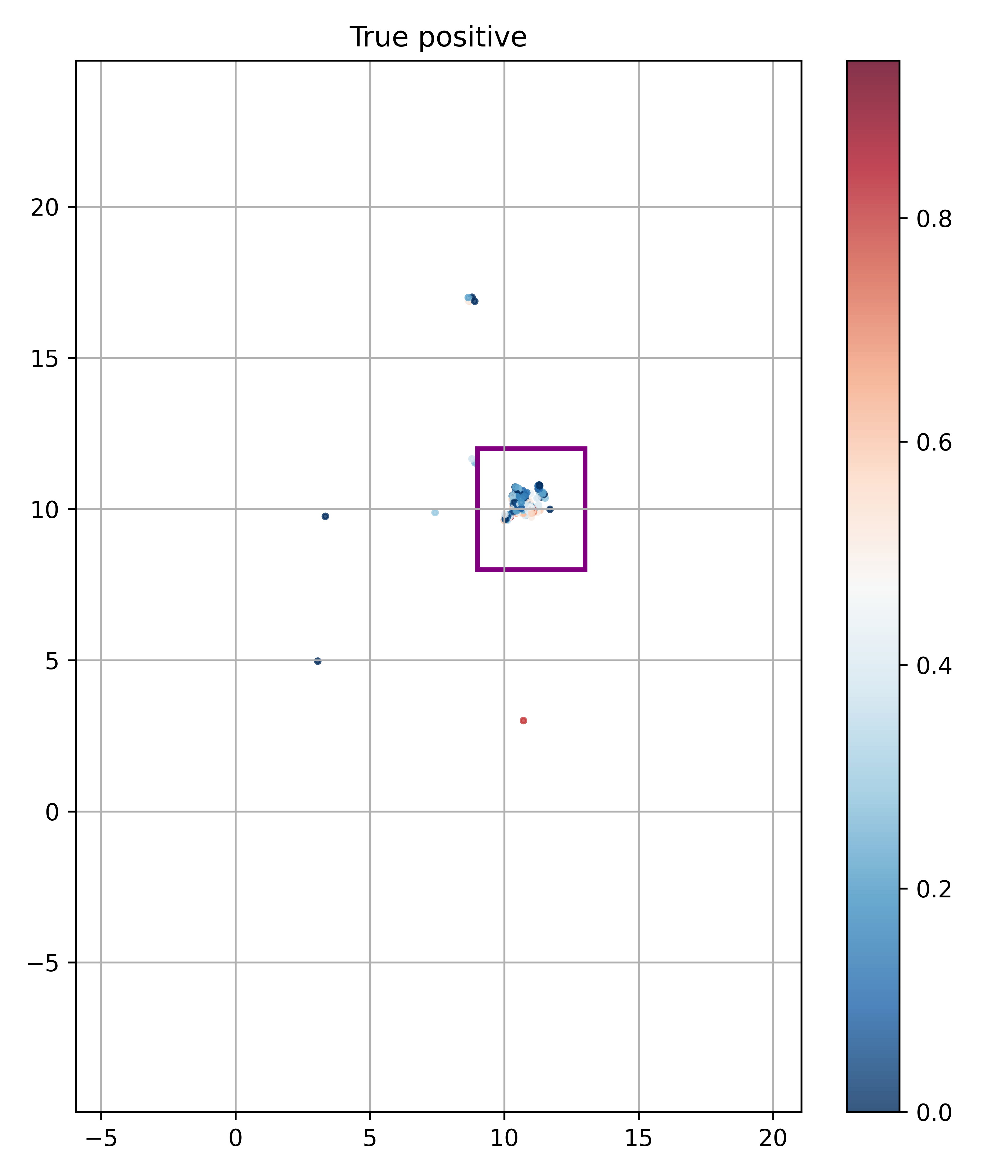

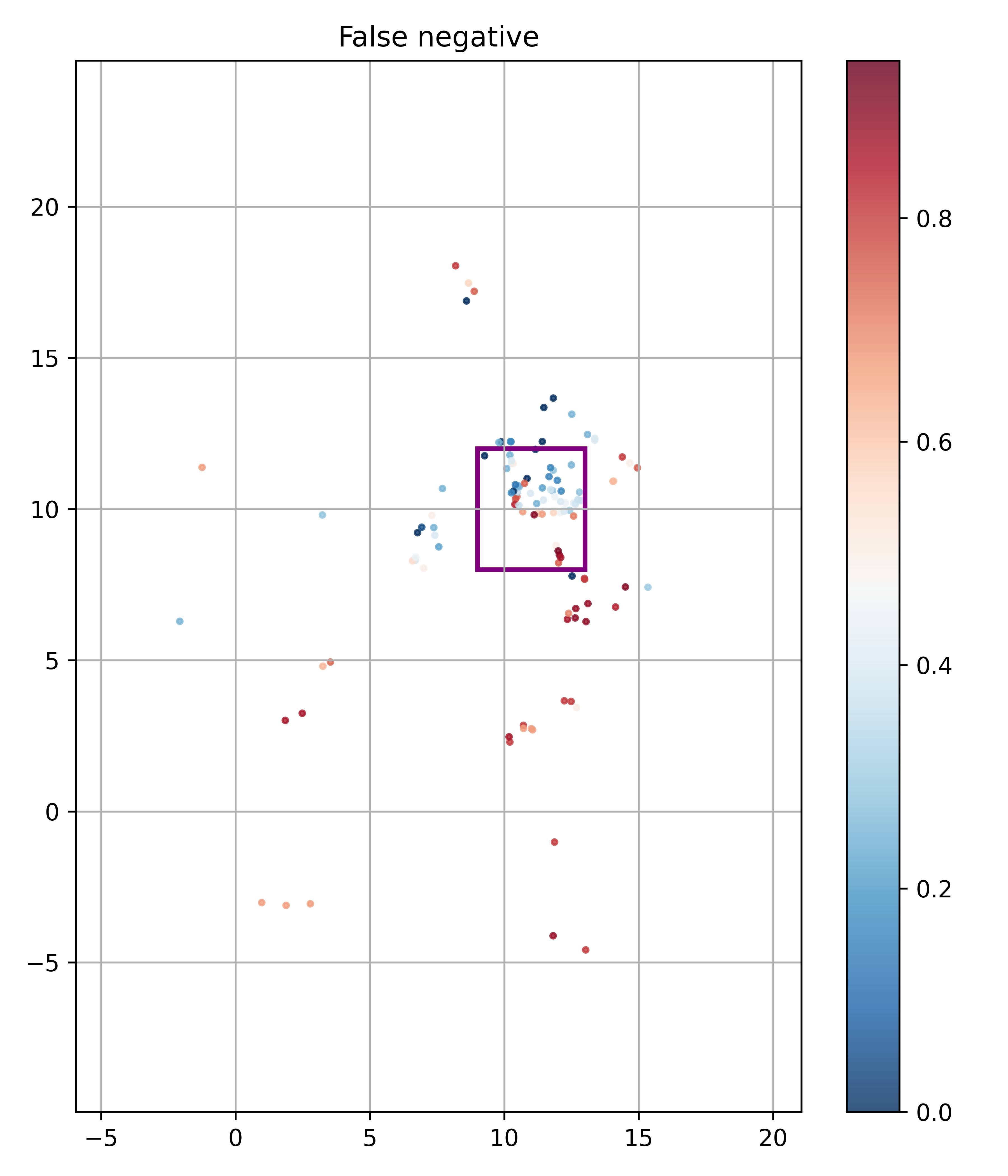

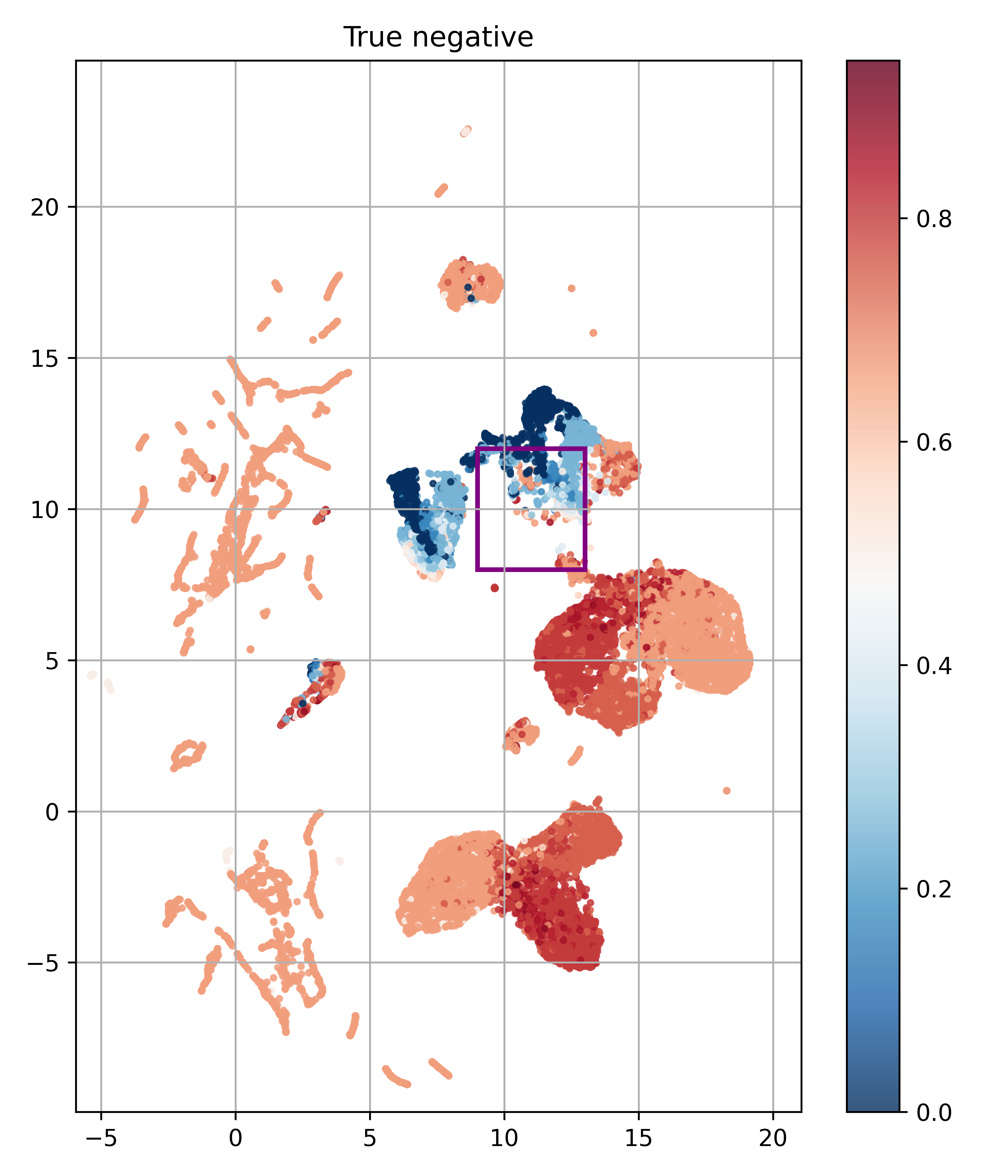

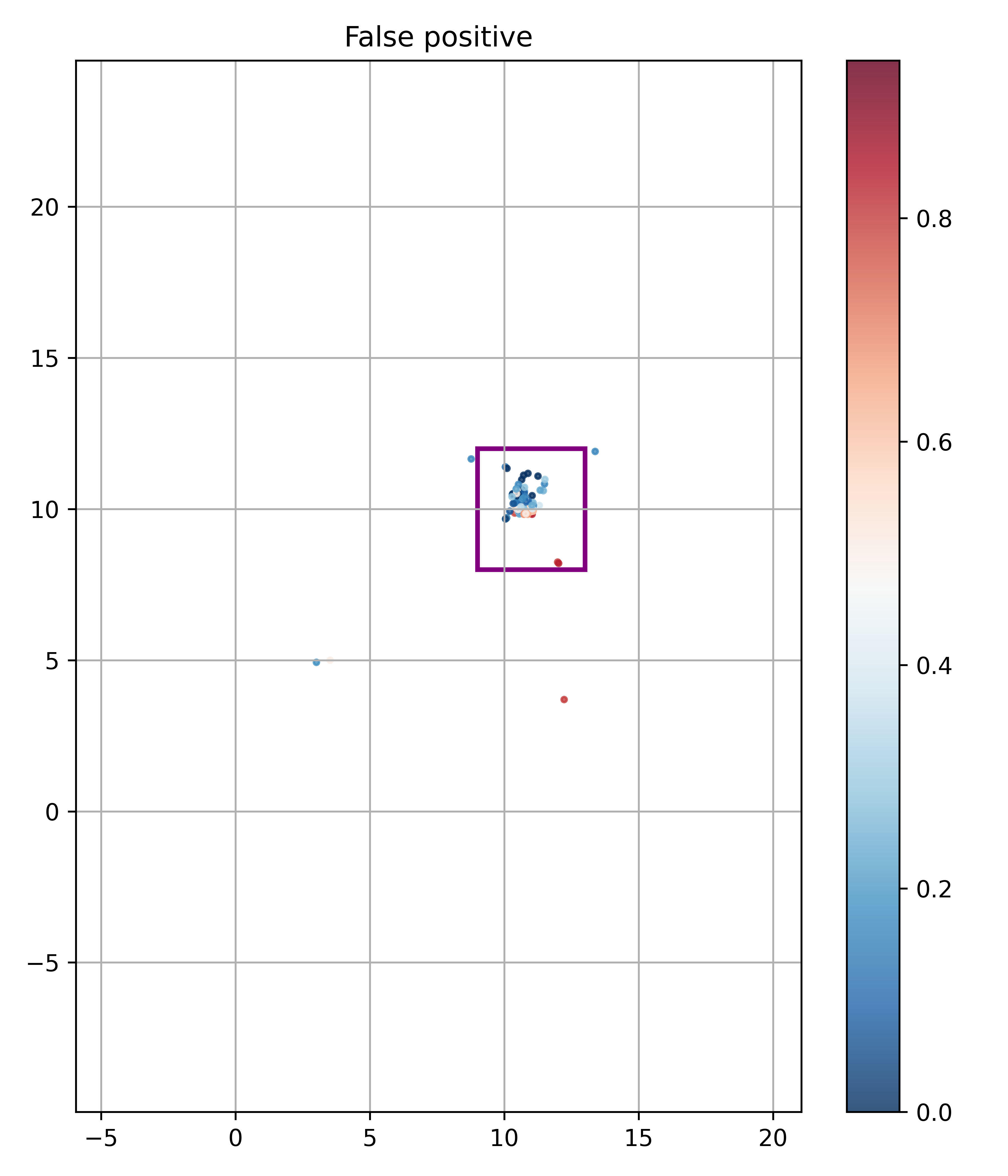


(D)

(C)

(A)

(B)

**Supplementary Fig. S4** UMAPs represent the outputs of the second hidden layer of DEEPOMICS^®^ FFPE for true FFPE-artifacts (A), false variants (B), false FFPE-artifacts (C), and true variants (D). The color scale represents SOB scores of a given variant. The purple rectangles indicate the areas where DEEPOMICS FFPE is mostly confident (Supplementary Fig. 3).


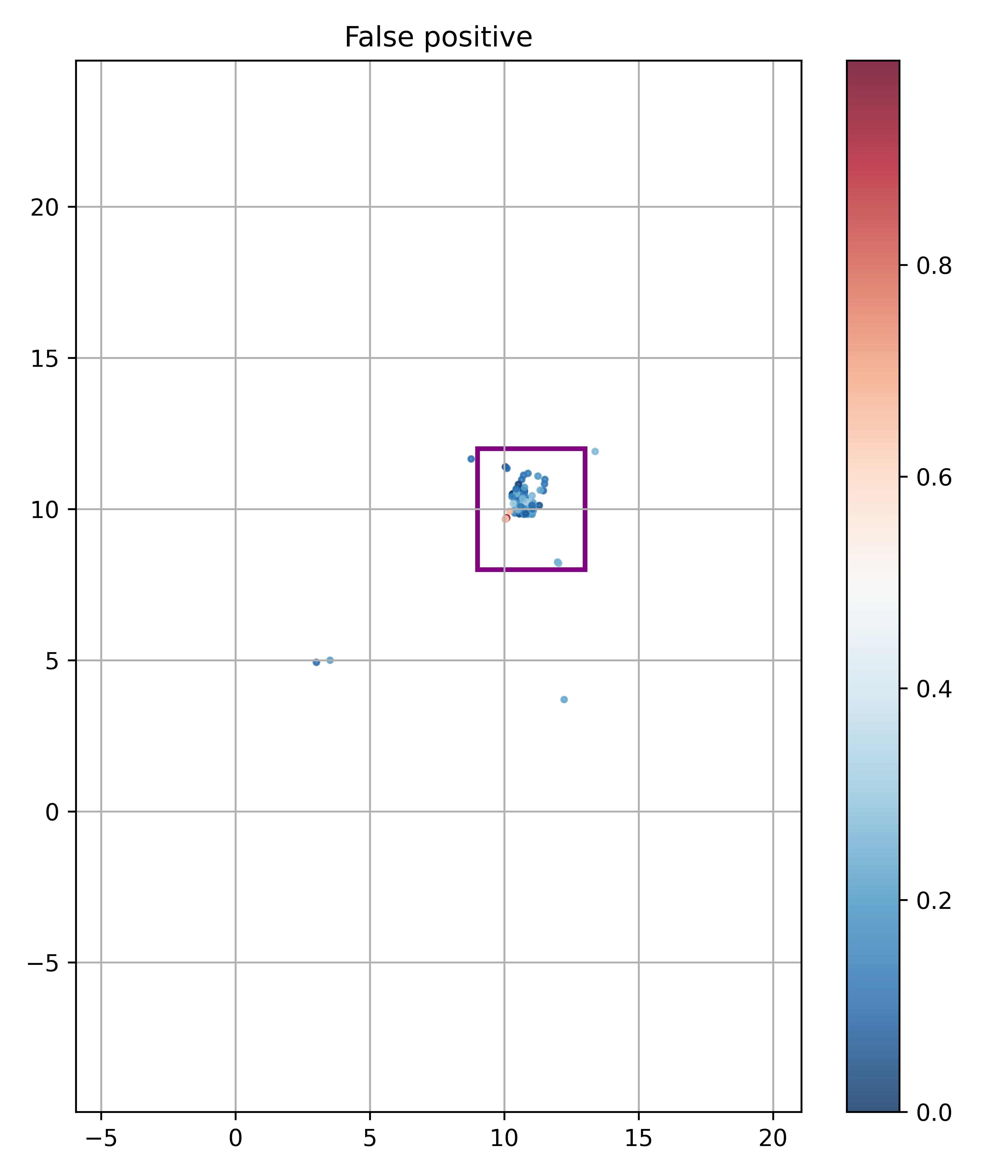

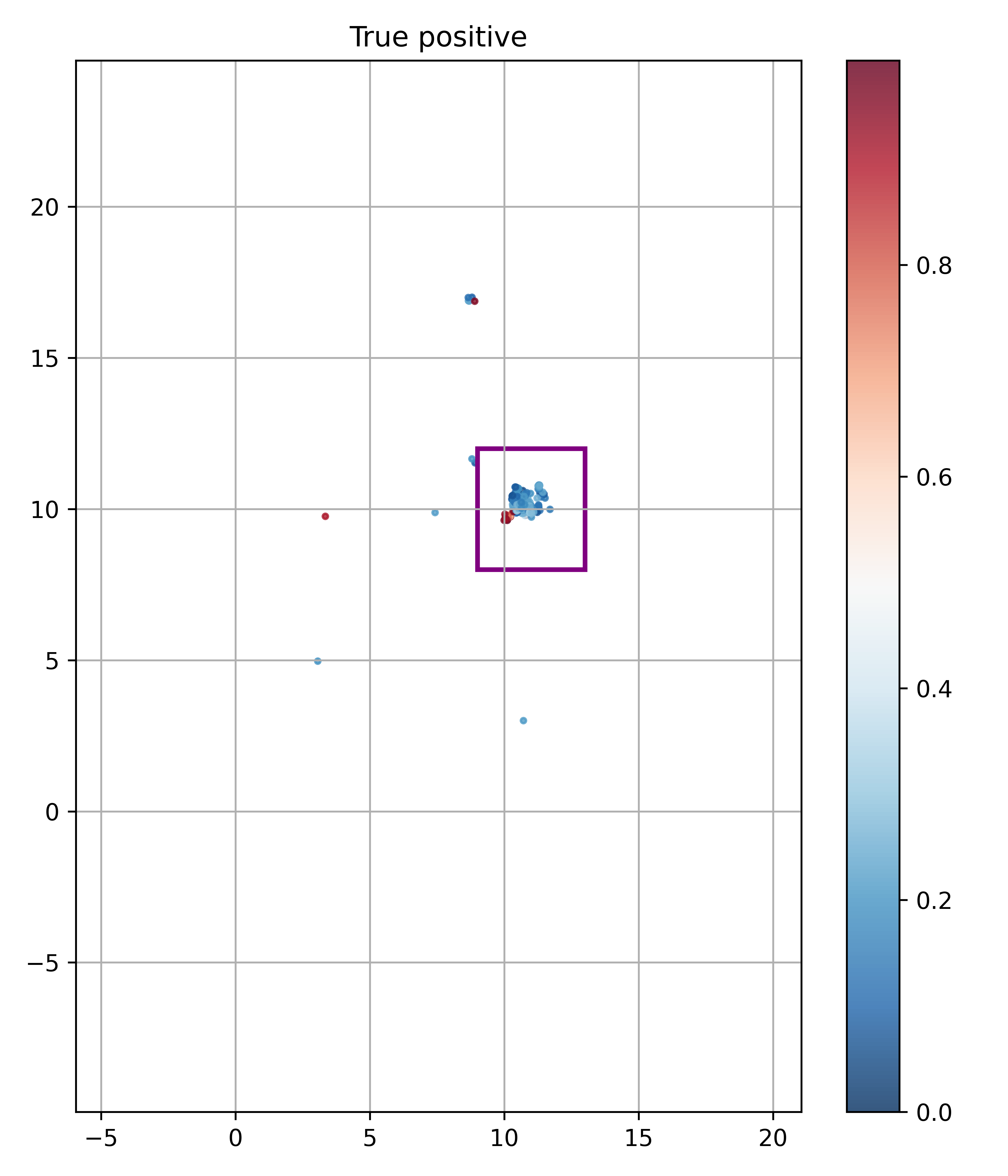

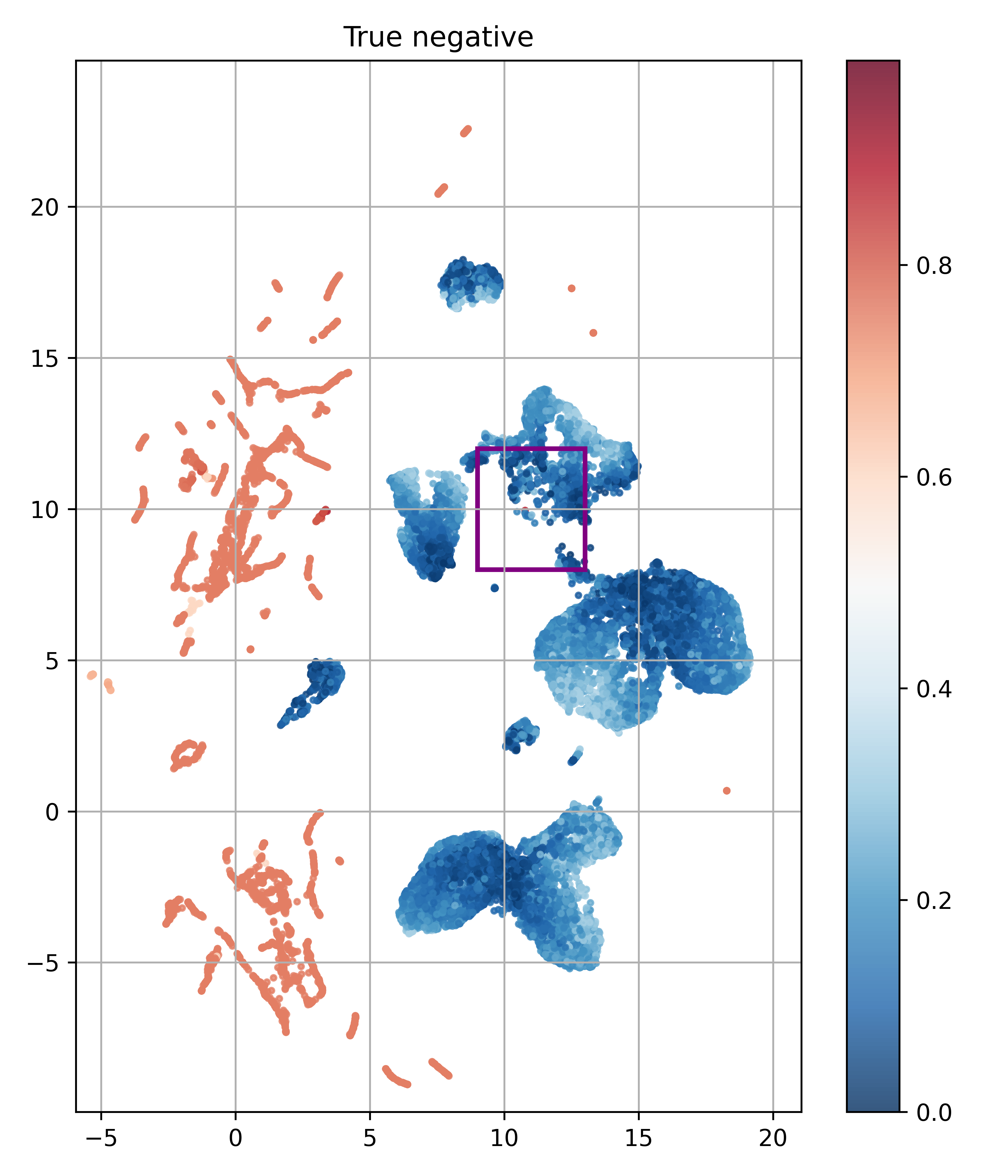

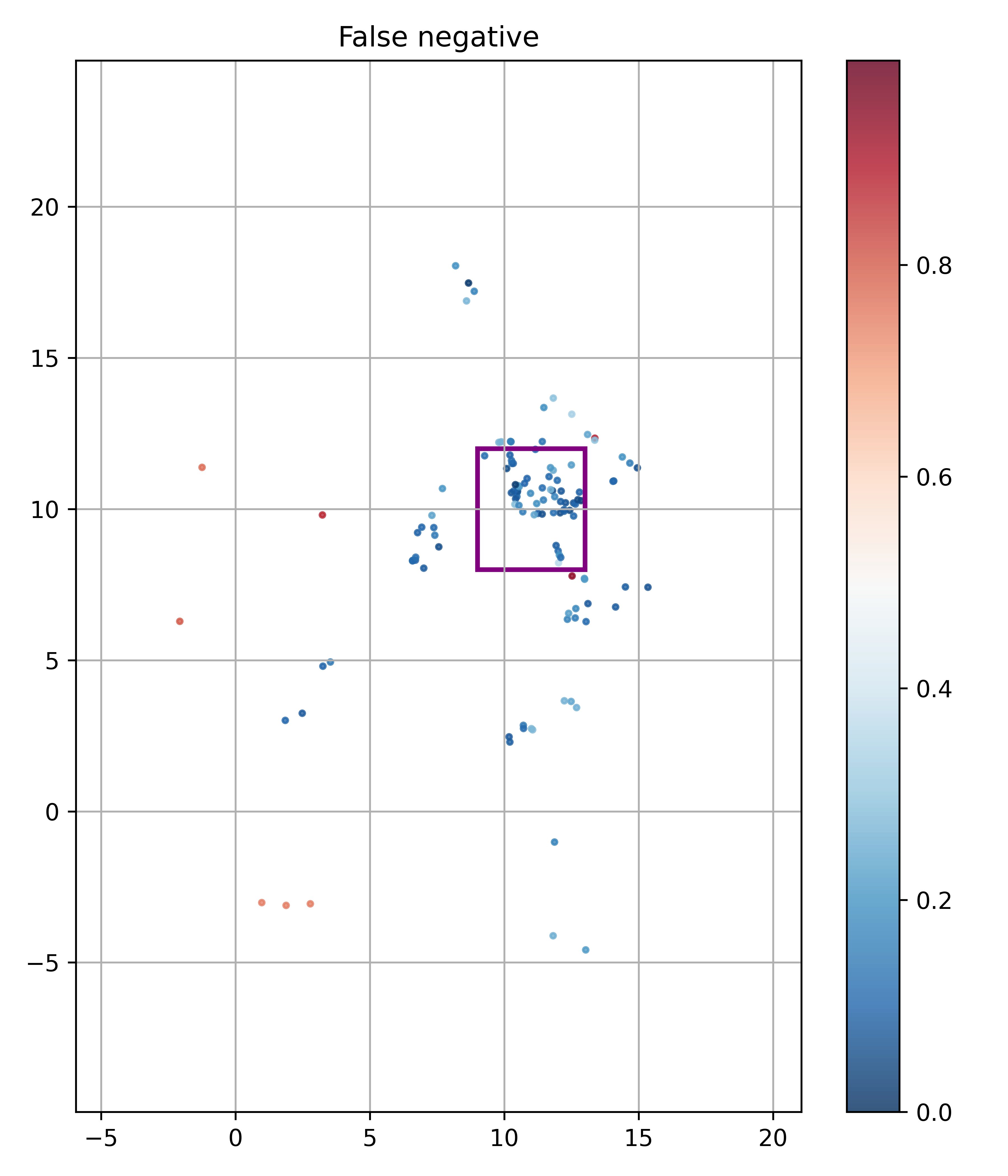


(D)

(C)

(A)

(B)

**Supplementary Fig. S5** UMAPs represent the outputs of the second hidden layer of DEEPOMICS^®^ FFPE for true FFPE-artifacts (A), false variants (B), false FFPE-artifacts (C), and true variants (D). The color scale represents MAF of a given variant. The purple rectangles indicate the areas where DEEPOMICS FFPE is mostly confident. (Supplementary Fig. 3).


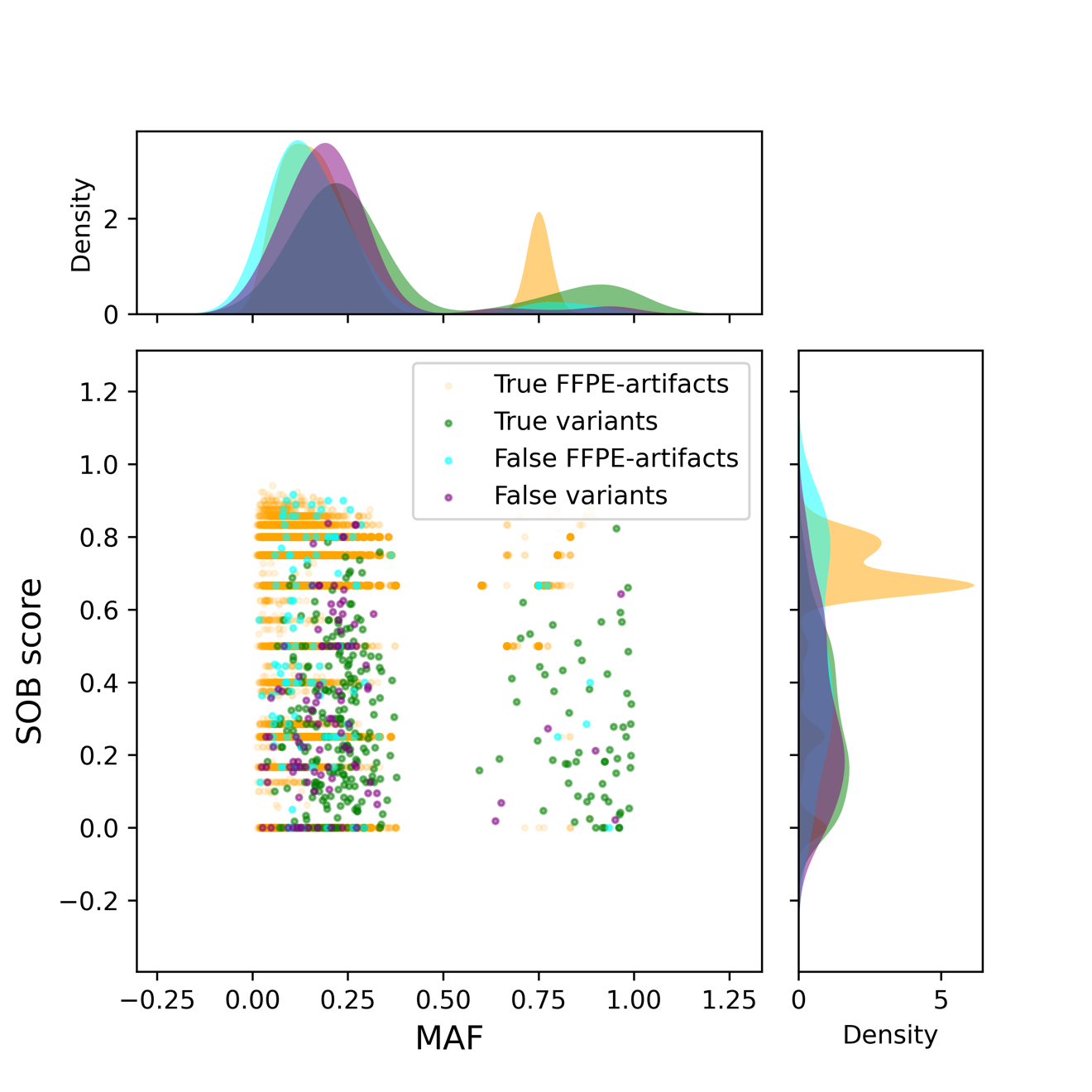


**Supplementary Fig. S6** MAF and SOB score of variants were plotted. True FFPE-artifacts (true negatives), true variants (true positives), false FFPE-artifacts (false negatives), and false variants (false positive) are indicated in orange, green, cyan, and purple, respectively. Density plots represent the distribution of MAF (upper panel) and SOB scores (right panel) of variants.


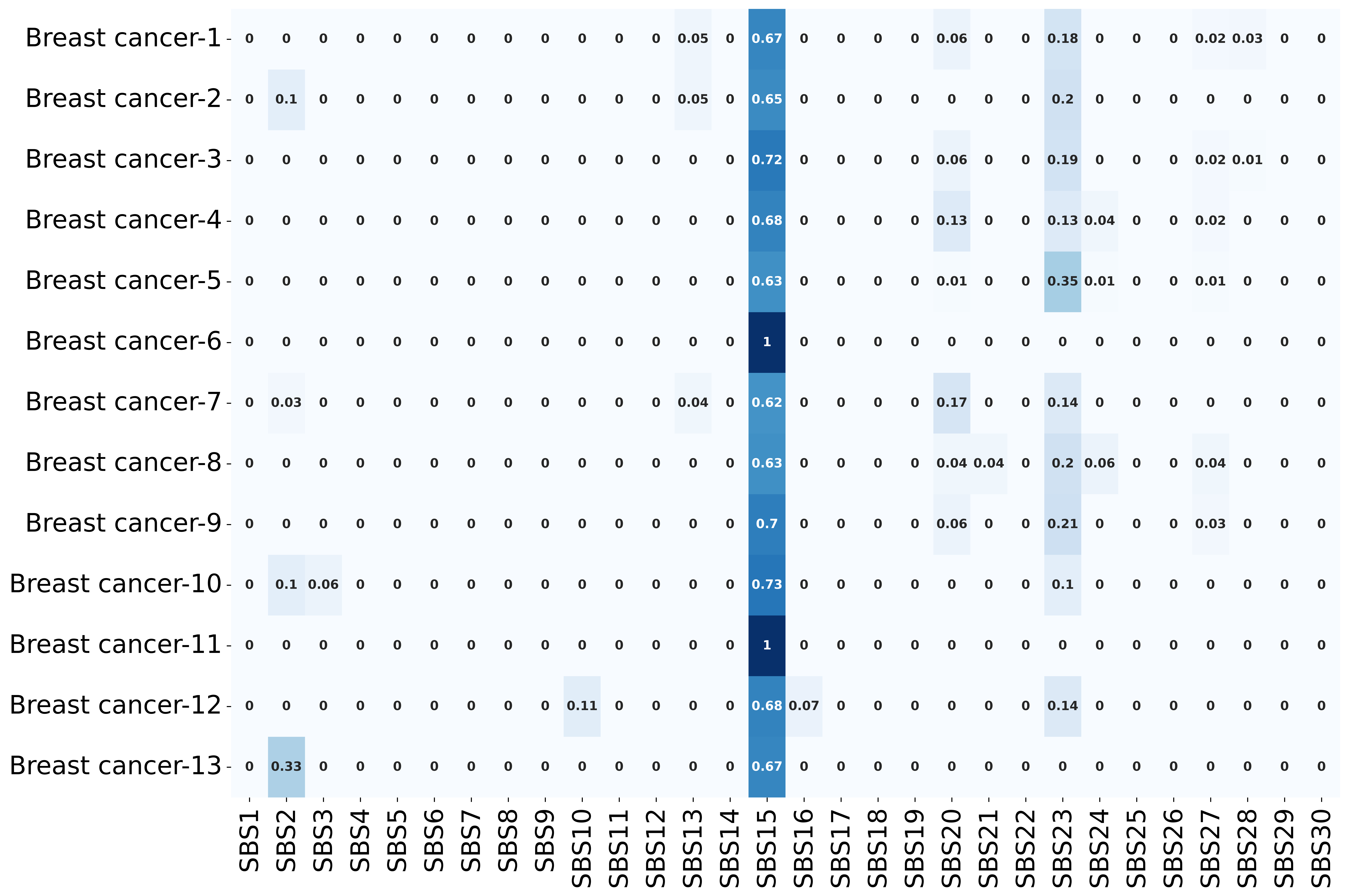


(A) FF


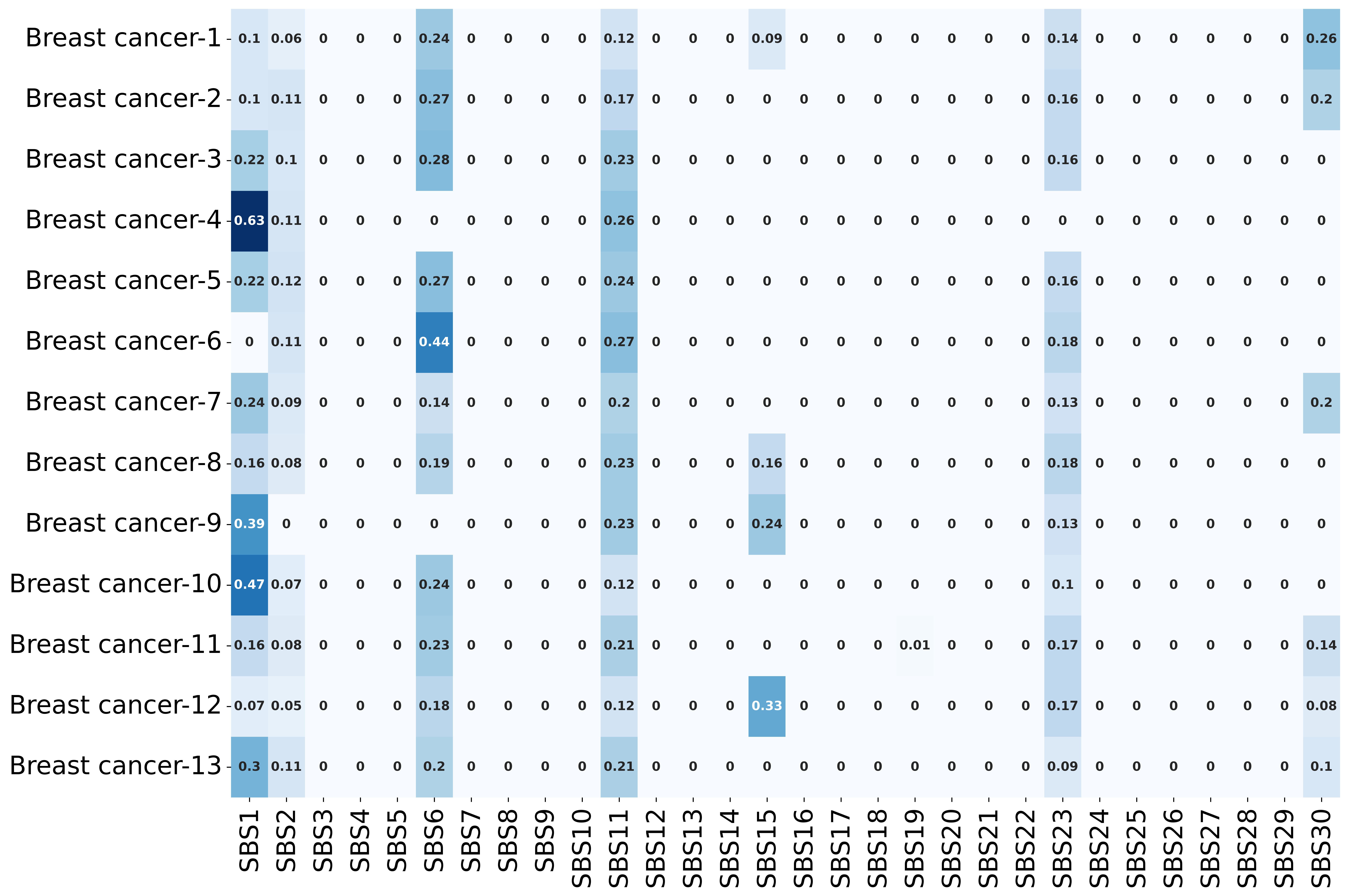


(B) FFPE (Before DEEPOMICS FFPE)


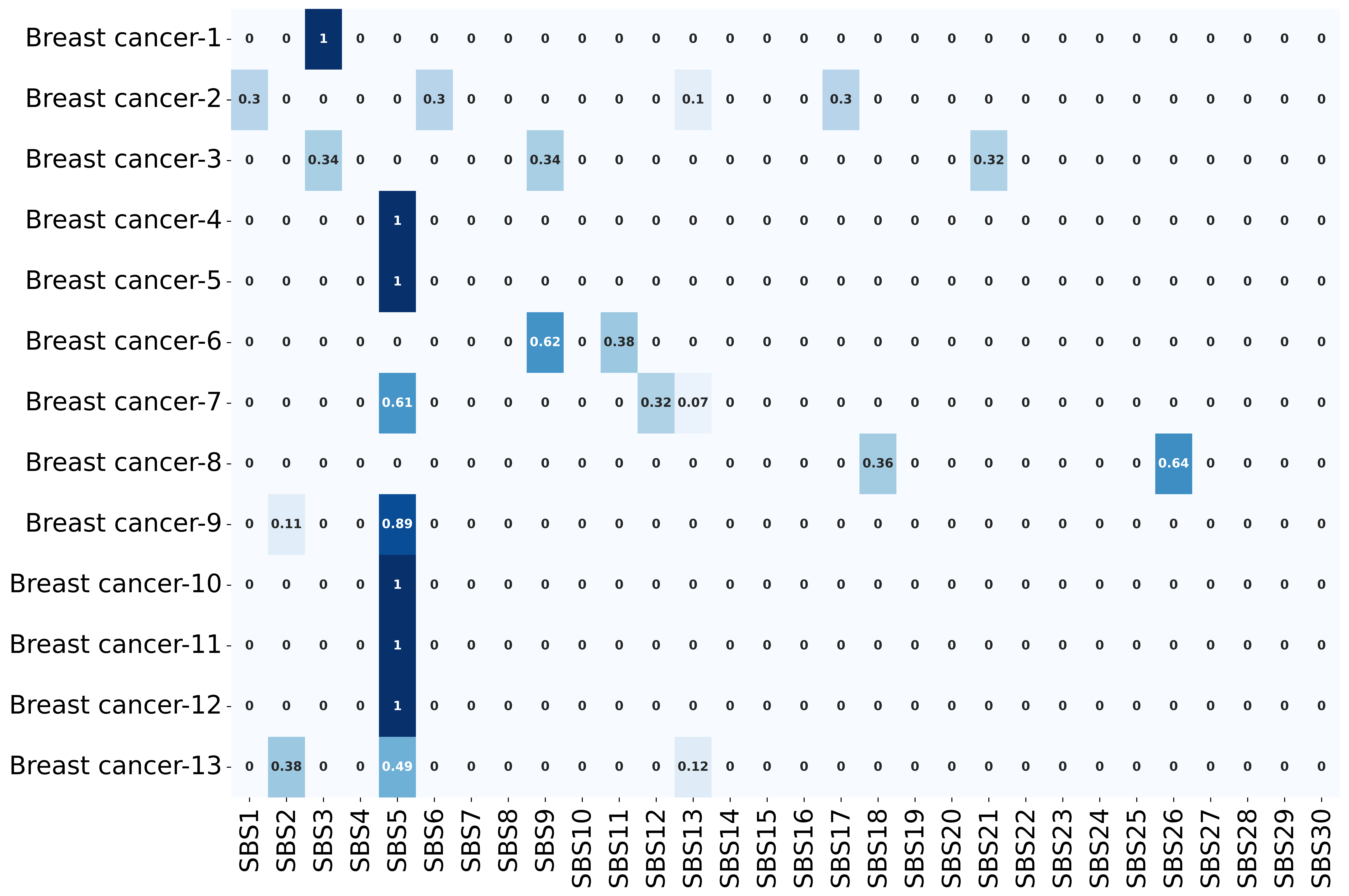


(C) FFPE (After DEEPOMICS FFPE)

**Supplementary Fig S7. Mutational signatures observed in breast cancer samples used in this study.**

Heatmap represents the contribution of the corresponding signature catalogs observed in 13 breast cancer samples.
